# Mechanically‐Gated Stochastic Resonance for All‐Optical Acoustic Sensing in Noisy Environments

**DOI:** 10.1002/advs.78634

**Published:** 2026-11-05

**Authors:** Xuyu Zhang, Minzheng Sun, Cunzheng Fan, Shuolong Zhu, Qilin Hong, Pan Xu, Min Zhu, Zhengliang Hu, Yongchao Zou, Qizhen Sun

**Affiliations:** ^1^ College of Meteorology and Oceanography National University of Defense Technology Changsha Hunan China; ^2^ National Engineering Research Center for Next Generation Internet Access System (NGIA) School of Optical and Electronic Information Huazhong University of Science and Technology Wuhan Hubei China

**Keywords:** acoustic sensing, fiber Bragg grating, optical fiber sensors, stochastic resonance

## Abstract

Weak signal detection in harsh and noisy environments remains a critical challenge across diverse fields. Stochastic resonance (SR) offers a counterintuitive strategy by exploiting noise to enhance, rather than suppress, weak signals, yet its practical deployment is severely limited by poor frequency selectivity, inadequate adaptability to fluctuating noise levels, and reliance on offline post‐processing. Here we report the discovery and experimental demonstration of mechanically‐gated stochastic resonance (MGSR), a novel physical mechanism that couples stochastic resonance with intrinsic mechanical resonance. By using mechanical resonance to actively modulate the bistable potential, MGSR achieves targeted frequency‐line selection, robust adaptation to variable noise conditions, and markedly improved signal‐to‐noise ratio (SNR) gain for weak signal detection. We further realize a real‐time, electromagnetic‐interference‐immune all‐optical bistable system through a fiber Bragg grating feedback mechanism, enabling hardware‐level MGSR free of computational latency. In tests conducted under realistic ocean ambient noise, our system attains a substantial SNR gain of 19.26 dB at the designed frequency, representing a clear advance for hardware‐based stochastic resonance acoustic sensors. This work deepens the understanding of SR, establishes a robust all‐optical architecture, and opens a scalable pathway toward high‐performance, noise‐resilient acoustic and vibration sensing for environmental monitoring, structural health diagnosis, and underwater safety.

## Graphical abstract

A mechanically‐gated stochastic resonance (MGSR) sensing system is demonstrated, wherein stochastic resonance is enabled by an all‐optical bistable fiber Bragg grating feedback loop and gated by mechanical resonance. Under ambient noise, the system generates bistable outputs satisfying the Kramers escape‐rate condition, yielding signal‐to‐noise ratio (SNR) gain for weak acoustic signals. This all‐optical, real‐time approach offers frequency‐selective, noise‐robust, electromagnetic‐interference‐immune detection.

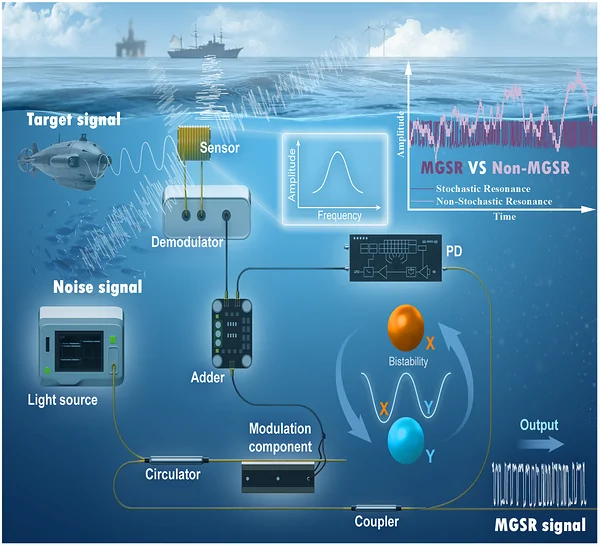

## 1 Introduction

Extracting weak signals from intense, non‐stationary noise backgrounds remains a persistent, cross‐disciplinary challenge encompassing environmental monitoring [1], structural health diagnosis [2], underwater safety [3], and aerospace engineering [4]. Taking underwater acoustic detection for example, target signatures primarily comprise line spectra, whose accurate extraction is critical for target identification [5, 6]. However, detection performance is increasingly compromised by two synergistic trends. First, advancements in vibration and noise reduction technologies, such as acoustic metamaterials [7], have attenuated target signal strengths to levels nearly indistinguishable from background noise [8]. Second, the proliferation of maritime shipping, resource exploration, and offshore engineering has escalated both the complexity and intensity of ocean ambient noise [9]. Consequently, conventional frameworks rely heavily on noise suppression strategies, such as adaptive cancellation [10], digital denoising [11], and spatial filtering [12]. These methods inevitably attenuate weak signals, depend on predefined priors that fail to generalize in non‐stationary environments, and incur computational latency that precludes real‐time operation, thereby imposing a performance ceiling on high‐sensitivity sensing.

In 1981, Benzi and colleagues first reported the stochastic resonance (SR) effect while investigating glacial cycles, revealing a counterintuitive mechanism whereby noise excitation enhances the detection of weak signals [13]. Traditionally, SR is realized in nonlinear systems and has been extensively investigated across diverse domains, including optomechanical resonators [14, 15], wearable monitoring [16, 17], MEMS sensing [18], nanoelectromechanical systems [19], microwave detection [20, 21], ferroelectric devices [22], structural health monitoring [2], and fluid sensing [23]. Beyond this classical nonlinear paradigm, a parallel line of research has emerged under the framework of generalized stochastic resonance (GSR), which broadens the concept of SR by identifying non‐monotonic response behaviors that depend on system and noise parameters, and demonstrates that SR‐like phenomena can also arise in linear systems driven by multiplicative noise [24, 25]. Recent studies have further extended GSR to practical fault diagnosis applications, including robust GSR arrays for multi‐harmonic feature enhancement under variable‐speed conditions [26], integration with lightweight neural networks for efficient classification [27], and GSR‐enhanced sparse feature learning under data‐scarce scenarios [28].

However, existing SR implementations predominantly rely on algorithmic post‐processing [29, 30, 31, 32, 33, 34, 35, 36, 37, 38], particularly in underwater acoustics [39, 40, 41, 42, 43, 44, 45, 46, 47, 48, 49, 50, 51, 52]. Moreover, when deployed in practical complex scenarios, these methods still suffer from three critical drawbacks. First, constrained by the transition frequency of the system operating point, they yield an SNR for low‐frequency signals after stochastic resonance processing that is consistently higher than that for high‐frequency signals under identical signal intensity, making them inherently biased toward low‐frequency components and lacking controllable frequency selectivity for targeted signal recovery. Second, their performance is severely compromised by wide‐range fluctuations in ambient noise intensity, leading to poor adaptability in dynamic environments. Third, their reliance on offline digital processing or electronic hardware introduces computational latency and vulnerability to electromagnetic interference [53, 54]. Consequently, existing SR methods have yet to achieve reliable, real‐time hardware‐level deployment in practical applications with harsh field environments.

In this work, we address this challenge by introducing and experimentally validating a novel physical mechanism that synergistically integrates stochastic resonance with the sensor's inherent mechanical resonance, which we term mechanically‐gated stochastic resonance (MGSR). By leveraging mechanical resonance to actively modulate the bistable potential landscape, MGSR achieves three critical advances over conventional approaches, namely targeted frequency‐selective enhancement of weak signals, robust adaptability to wide‐range dynamic noise environments, and an all‐optical hardware implementation that ensures real‐time response free from computational latency and electromagnetic interference. This mechanism effectively addresses the critical bottlenecks of both traditional noise suppression and existing SR systems, providing a scalable approach for high‐performance weak‐signal detection in harsh environments. To validate the MGSR mechanism, we constructed an all‐optical sensing system based on fiber Bragg gratings and systematically characterized its frequency selectivity, noise adaptability, and real‐time response. Experiments conducted under authentic ocean ambient noise demonstrate a substantial SNR gain of 19.26 dB at the designed frequency, representing a clear advance for hardware‐based SR acoustic sensors. This work deepens the understanding of nonlinear stochastic dynamics and provides a robust, scalable platform for next‐generation sensing in complex and demanding scenarios.

## 2 Results

### 2.1 Principle of Mechanically‐Gated Stochastic Resonance Mechanism

Stochastic resonance refers to the phenomenon where the interaction between noise and a periodic driving force in a nonlinear system leads to regular transitions in the system's output state [55]. The bistable output induced by changes in the operating point within nonlinear system is a classic example of stochastic resonance. Normally, a bistable system can be described by the first‐order Langevin equation [39] (Definitions of key variables and symbols and explanations of specialized terms used in the main text and supplementary materials are provided in Table S1):

(1) $$\frac{dx}{dt}=-\frac{dU\left(x\right)}{dx}+S\left(t\right)+N\left(t\right)$$

where *x* is the system operating point position, *U*(*x*) is the bistable potential curve, *S*(*t*) is the weak periodic excitation signal, and *N*(*t*) is the noise signal. Furthermore, *U*(*x*), *S*(*t*), and *N*(*t*) can be expressed as [56]:

(2) $$\left\{\begin{array}{c} U\left(x\right)=-a\cdot \frac{x^{2}}{2}+b\cdot \frac{x^{4}}{4},\left(a>0,b>0\right)\\ S\left(t\right)=A_{0}\cdot \cos\left(2\pi f\cdot t+\phi \right)\\ \langle N(t)\rangle =0,\langle N(t)N(0)\rangle =2D\cdot \delta \left(t\right) \end{array}\right.$$

Among these, *a* and *b* are the bistable system parameters, generally treated as constants, *A*_0_ is the amplitude of the periodic excitation signal, *f* is the signal frequency, *ϕ* is the total initial phase variation, *N*(*t*) represents Gaussian white noise, 〈*N*(*t*)〉 is the mean value of *N*(*t*), 〈*N*(*t*)*N*(0)〉 is the autocorrelation function of *N*(*t*), *D* is the noise intensity, and *δ*(·) denotes the Dirac delta function, indicating that the noise values at different times are uncorrelated. In Equation (1), the bistable potential curve *U*(*x*) with the negative sign signifies that the operating point is confined within a specific potential well. In the absence of noise *N*(*t*), the weak signal *S*(*t*) is insufficient to drive the operating point across the potential barrier. As a result, the operating point tends to undergo back‐and‐forth periodic motion within a single potential well. Consequently, the externally introduced noise signal becomes a critical factor enabling the operating point to surmount the potential barrier and transition between the two wells. By differentiating *U*(*x*) in Equation (2), it can be determined that when its derivative is zero, the potential wells and barrier of the bistable curve are obtained. Since *S*(*t*) is assumed to be a weak signal, its modulation effect on the potential landscape is negligible. In this static limit, the two potential wells and the barrier are located at $x_{1}=-\sqrt{a/b}$, $x_{3}=\sqrt{a/b}$, and *x*_2_ = 0, respectively (see Note S1). Substituting these solutions into the original equations, the static barrier height Δ*U*_0_ can be obtained as:

(3) $$\Delta U_{0}=\frac{a^{2}}{4b}$$

Based on the analysis of Equation (1), it can be seen that when the externally applied noise signal *N*(*t*) is stronger than the potential barrier height Δ*U*_0_, the system's operating point can cross the potential barrier and move back and forth between the two potential wells, thereby producing a bistable output. However, it should be noted that a bistable output does not necessarily imply the stable occurrence of stochastic resonance. Combining Equations (1) and (2), it can be found that to optimize the effect of stochastic resonance, the transition effect of the system's operating point between the two potential wells must satisfy a statistical relationship as well. The Kramers escape rate describes the rate at which the operating point, driven by noise, escapes from a potential well in a single event, and this relationship can be expressed as [56]:

(4) $$r_{k}=\frac{\sqrt{2}a}{2\pi }\exp\left(-\frac{a^{2}}{4bD}\right)$$

From Equation (4), it can be found that the escape rate *r_k_* depends only on the noise intensity *D*. Based on the concept of the Kramers escape rate, the effect of stochastic resonance is optimized under the condition *r_k_* = 2*f*. By combining the two expressions for *r_k_*, the optimal noise intensity corresponding to different signal frequencies is:

(5) $$D=\frac{a^{2}}{4b\cdot \ln\left(\frac{\sqrt{2}a}{4\pi f}\right)}$$

However, the theoretical model described above is established under the assumption that both the amplitude *A*_0_ and frequency *f* of the input signal are relatively small [57]. In practical applications, these conditions are not always satisfied. In particular, when the amplitude of the external signal undergoes nonlinear gain due to mechanical resonance, the bistable output will transition from being determined solely by the noise intensity *D* to being jointly regulated by both *D* and the periodic excitation signal amplitude *A*_0_. Under such circumstances, the effect of the periodic excitation signal on the bistable potential function cannot be neglected, and the static assumption of the bistable potential function no longer holds. Consequently, we need to further deepen Equation (2) and, on this basis, generalize the Kramers escape rate model. According to the derivation in Note S1, the instantaneous bistable potential function can be expressed as [55]:

(6) $$U\left(x,t\right)=-\frac{a}{2}x^{2}+\frac{b}{4}x^{4}-S\left(t\right)\cdot x$$

From the above equation, it is evident that the introduction of the linear tilt term −*S*(*t*)·*x* imposes a time‐periodic alternating tilting effect on the static bistable potential *U*_0_(*x*), causing dynamic shifts in the positions of the potential wells and barrier, and rendering the barrier height no longer constant. By differentiating Equation (6), we obtain the cubic equation in *x* as:

(7) $$bx^{3}-ax-S\left(t\right)=0$$

From its discriminant Δ(*t*) (see Note S1), it can be seen that when Δ(*t*) > 0, the instantaneous bistable potential function has two potential wells *x*_1_(*t*) and *x*_3_(*t*) and a barrier *x*_2_(*t*), with *x*_1_(*t*) < *x*_2_(*t*) < *x*_3_(*t*). When Δ(*t*) = 0, the bistable barrier vanishes, and the instantaneous amplitude *A_c_* of the periodic excitation signal *S*(*t*) is [58]:

(8) $$A_{c}=\sqrt{\frac{4a^{3}}{27b}}$$

Combining with *A_c_*, the time‐dependent barrier height under periodic driving is expressed as:

(9) $$\Delta U\left(t\right)=\Delta U_{0}\cdot {\left\{1-{\left[\frac{S(t)}{A_{c}}\right]}^{2}\right\}}^{\frac{3}{2}}$$

Which reduces to Δ*U*_0_ when *S*(*t*) = 0, and vanishes when |*S*(*t*)| = *A_c_*. Combining the second derivative of Equation (6) with respect to *x* and Equation (9), we obtain the expression for the instantaneous Kramers escape rate as [56, 59]:

(10) $$r_{k}\left(t\right)=\frac{1}{2\pi }\sqrt{U^{\prime \prime }\left[x_{1}\left(t\right)\right]\left|U^{\prime \prime }\left[x_{2}\left(t\right)\right]\right|}\exp\left[-\frac{\Delta U(t)}{D}\right]$$

Combining with the previous *r_k_* = 2*f*, we obtain the instantaneous optimal noise as:

(11) $$D_{opt}\left(t\right)=\frac{\Delta U(t)}{\ln\left[\frac{\sqrt{U^{\prime \prime }\left[x_{1}\left(t\right)\right]\left|U^{\prime \prime }\left[x_{2}\left(t\right)\right]\right|}}{4\pi f}\right]}$$

However, it should be noted that the escape process is not uniformly distributed across all moments. Because the Kramers escape rate depends exponentially on the barrier height, escape mainly occurs at the instant when the barrier is lowest, that is, when the periodic excitation signal reaches its maximum amplitude. Therefore, in the subsequent derivations, we default to using the maximum instantaneous amplitude of *S*(*t*) to represent this most probable transition instant, rather than the time‐varying *S*(*t*).

Physically, this maximum amplitude is determined by the sensor's mechanical response. When mechanical resonance is present, the received signal amplitude becomes frequency‐dependent, defined as *A_eff_* (*f*) [60]:

(12) $$A_{0}\equiv A_{eff}\left(f\right)=A_{r}\cdot \exp\left[-\frac{{\left(f-f_{c}\right)}^{2}}{2w^{2}}\right]$$

where *A_r_* denotes the maximum response amplitude of the sensor at the resonant frequency, *f* is the current input signal frequency, *f_c_* is the resonant frequency, and *w* is the standard deviation of the Gaussian approximation. This Gaussian form is a near‐resonance approximation to the intrinsic frequency response of sensor (see Note S11). Substituting Equation (12) into Equations (10) and (11) in sequence, we obtain the Kramers escape rate under mechanical resonance conditions and the optimal relationship between noise intensity and signal frequency as:

(13) $$\left\{\begin{array}{c} r_{k}\left[A_{eff}\left(f\right)\right]=\frac{1}{2\pi }\sqrt{U^{\prime \prime }\left\{x_{1}\left[A_{eff}\left(f\right)\right]\right\}\left|U^{\prime \prime }\left\{x_{2}\left[A_{eff}\left(f\right)\right]\right\}\right|}\\ \;\exp\left\{-\frac{\Delta U\left[A_{eff}\left(f\right)\right]}{D}\right\}\\ D_{opt}\left(f\right)=\frac{\Delta U\left[A_{eff}\left(f\right)\right]}{\ln\left\{\frac{\sqrt{U^{\prime \prime }\left\{x_{1}\left[A_{eff}\left(f\right)\right]\right\}\left|U^{\prime \prime }\left\{x_{2}\left[A_{eff}\left(f\right)\right]\right\}\right|}}{4\pi f}\right\}} \end{array}\right.$$

It can be seen that when *A_eff_* (*f*) = 0, Equation (13) reduces to Equation (4) and Equation (5). This indicates that the generalized Kramers escape rate theoretical model can be applied to predict the output of stochastic resonance under both small‐parameter and non‐small‐parameter conditions, providing a theoretical foundation for subsequent simulation tests and experimental validation. Further details regarding the generalized Kramers escape rate theory model can be found in Note S2. On this basis, we define the effective barrier height modulation factor *µ*, which is expressed as the ratio of the tilted barrier to the static barrier:

(14) $$\mu \left(f\right)=\frac{\Delta U\left[A_{eff}\left(f\right)\right]}{\Delta U_{0}}={\left\{1-{\left[\frac{A_{eff}\left(f\right)}{A_{c}}\right]}^{2}\right\}}^{\frac{3}{2}}$$

At this point, *µ* can be regarded as a barrier tunable gate. By adjusting the magnitude of *µ*, the processing effect of stochastic resonance can be effectively modulated. Under the same mechanical structure with *A_c_* taken as a constant, the value of *µ* is primarily influenced by the mechanical resonant response. Therefore, the above theory is referred to as mechanically‐gated stochastic resonance (MGSR). Utilizing the properties of MGSR, it is expected to overcome the limitations currently faced by stochastic resonance sensing. It should be noted that the preceding theoretical derivation only involves the processing of the periodic excitation signal to be measured by MGSR. Detailed derivations concerning the integration of MGSR theory with practical sensing applications can be found in Notes S1, S3, and S11.

### 2.2 Simulation Validation of MGSR Effect

To substantiate the theoretical foundation of MGSR, we established a numerical model based on the generalized Kramers escape rate, incorporating mechanical resonance as a gating mechanism. The model parameters, specifically the standard deviation of the mechanical resonance profile *w*, maximum response amplitude *A*_0_, and resonant frequency *f_c_*, were treated as variables, with the noise intensity *D* in the numerical simulation scaled by standard normal distributions with a mean of 0 and a variance of 1. Following the standard dimensionless transformation for the bistable system, the dimensionless state variable *x*, noise intensity *D*, and driving amplitude *A*_0_ are defined by scaling with the characteristic length $\sqrt{a/b}$, the static barrier height Δ*U*_0_, and the critical amplitude *A_c_*, respectively [61]. In the MGSR framework, these dimensionless quantities are defined as:

(15) x∼=xaabb,D∼=DΔU0,A∼0=A0Ac

These dimensionless quantities directly capture the competition among the signal, the noise, and the nonlinear potential. Figure 1a contrasts a 60 Hz target signal with an amplitude of 0.232 submerged in noise ($\tilde{D}$= 0.8) against the reconstructed bistable output (Parameter configurations for the subsequent simulations and experiments are provided in Table S2). The periodic characteristics, initially indiscernible in the raw signal, are recovered via stochastic resonance, confirming the model's fidelity. Subsequently, we quantified the SNR gain across varying noise intensities ($\tilde{D}$∈ [0.08,1.6]) for signals at 60 Hz with amplitudes of 0.046, 0.093, and 0.232, as shown in Figure 1b. Unlike the monotonic SNR decay observed in traditional untreated signals, the SR‐processed output exhibited a characteristic non‐monotonic curve. As the noise intensity increases, the transition rate of the working point of the stochastic resonance system and the signal frequency gradually satisfy *r_k_* = 2*f*, consequently leading to a gradual increase in the SNR after SR processing. Figure 1c shows that the SNR gain increases exponentially with $\tilde{D}$, transitioning from negative to positive values beyond $\tilde{D}$= 0.176. Crucially, reducing the signal amplitude from 0.232 to 0.046 elevated the peak gain from 8.01 to 10.44 dB, elucidating SR's capacity for weak‐signal amplification.

**FIGURE 1:**
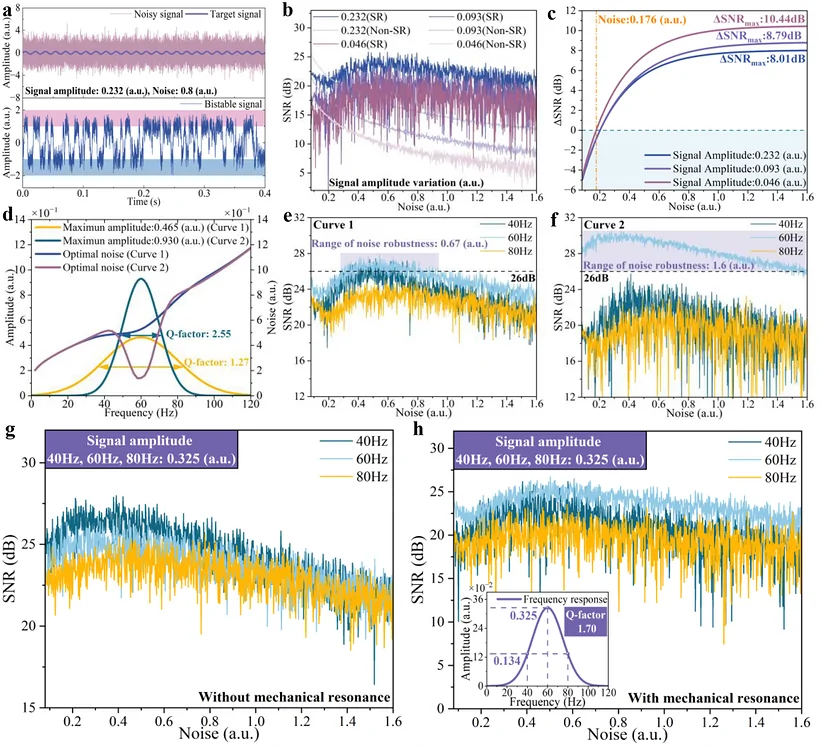
Simulation of the MGSR. (a) Time domain comparison among the original noisy signal (mixture of target signal and noise) and the bistable output signal after stochastic resonance processing. (b) Comparison of SNR curves with and without stochastic resonance processing under different signal amplitudes. (c) Stochastic resonance gain curves under different signal amplitudes. (d) Frequency response curves under mechanical resonance and the influence of frequency response curves on the optimal noise curve in stochastic resonance. (e) Distribution of SNR curves after stochastic resonance processing for signals of different frequencies under frequency response Curve 1. (f) SNR curves after stochastic resonance processing for signals of different frequencies under frequency response Curve 2. (g) Distribution of SNR curves after simultaneous stochastic resonance processing for signals of different frequencies without mechanical resonance. (h) Distribution of SNR curves after simultaneous stochastic resonance processing for signals of different frequencies in the presence of mechanical resonance. The inset shows the current frequency response curve distribution.

Subsequently, we introduced mechanical resonance into the existing SR system model to verify the reliability of the MGSR theory. Without loss of generality, two mechanical resonance curves (Curve 1 and Curve 2) with quality factors (Q‐factor) of 1.27 and 2.55 and peak values of 0.465 and 0.930 at 60 Hz, respectively, were incorporated into the model, as shown in Figure 1d. The resulting SNR curves for target signals at 40, 60, and 80 Hz are presented in Figure 1e. It can be observed that when Curve 1 is introduced, the SNR curve for the 60 Hz signal is higher than those for the other two frequencies, in contrast to the monotonic decreasing trend of SNR with increasing signal frequency observed in conventional SR systems without mechanical resonance (Figure S1a). Notably, MGSR systems exhibit a distinct frequency‐selective characteristic, representing a critical advance for underwater acoustic detection. To further evaluate frequency selectivity under multi‐tone excitation, we subjected the system to concurrent sinusoidal inputs at 40, 60, and 80 Hz. In the absence of mechanical resonance, the SNR curves in Figure 1g exhibit a monotonic decline with increasing frequency, indicating that conventional stochastic resonance inherently favors low‐frequency components. Upon activating mechanical resonance with a Q‐factor of 1.70, as depicted in Figure 1h, the 60 Hz target signal experiences substantial nonlinear gain, elevating its amplitude to 0.325 and its SNR above those of off‐resonant frequencies. Furthermore, considering the prevalence of detection thresholds in practical systems, we adopted a SNR threshold of 26 dB as an example. Under this constraint, the 60 Hz signal supported a noise robustness range of 0.67 in Figure 1e. significantly exceeding that of off‐resonant frequencies, demonstrating MGSR's superior noise tolerance.

To elucidate the impact of mechanical resonance strength, we integrated Curve 2 exhibiting a higher Q‐factor into the system while maintaining an input amplitude of 0.930. As shown in Figure 1f, this modification yields two critical enhancements. First, the noise robustness range for the 60 Hz signal escalates from 0.67 to 1.6. substantially broadening noise tolerance. Second, off‐resonant frequencies at 40 and 80 Hz experience amplitude suppression, preventing their SNR from reaching the detection threshold. This selective gating arises from the dip structure in the optimal noise curve at resonance, as illustrated in Figure 1d and Figure S1b. At resonance, the required noise intensity for stochastic resonance is minimized, lowering the energy barrier for the target signal. Consequently, the synergy between this lowered barrier and the mechanical nonlinear gain confers superior noise immunity, validating MGSR's capability for targeted signal extraction in intense noise environments.

In summary, these simulations collectively substantiate the feasibility of MGSR by demonstrating its capacity for weak‐signal amplification, broadband noise robustness, and selective extraction of target frequencies.

### 2.3 Materialization of the MGSR Based on the Fiber Bragg Grating (FBG) Feedback Mechanism

Bistability is fundamental to realizing stochastic resonance. We therefore engineered a hardware stochastic resonance system utilizing a fiber Bragg grating (FBG) feedback mechanism, as depicted in Figure 2a, to yield bistable output. Specifically, an optical signal satisfying the Bragg condition is reflected and bifurcated into two paths. One path serves as the system output, while the other is converted to an electrical signal. This electrical signal is superimposed onto the external target signal and noise, subsequently modulating the FBG's central wavelength to establish a negative feedback loop. Within this bistable potential, the operating point resides in one of two wells. As illustrated in Figure 2a inset a_1_, a weak periodic signal alone cannot overcome the potential barrier. However, the introduction of sufficient external noise, such as that generated by offshore wind turbines or vessels, enables the operating point to transit between wells X and Y. When the transition rate satisfies the Kramers condition *r_k_* = 2*f*, the output frequency reconstructs the target signal, manifesting stochastic resonance. Furthermore, as shown in inset a_2_, the sensor's intrinsic mechanical resonance response integrates with this optical bi‐stability. This synergy constitutes the core mechanism of MGSR, enabling frequency‐selective signal enhancement.

**FIGURE 2:**
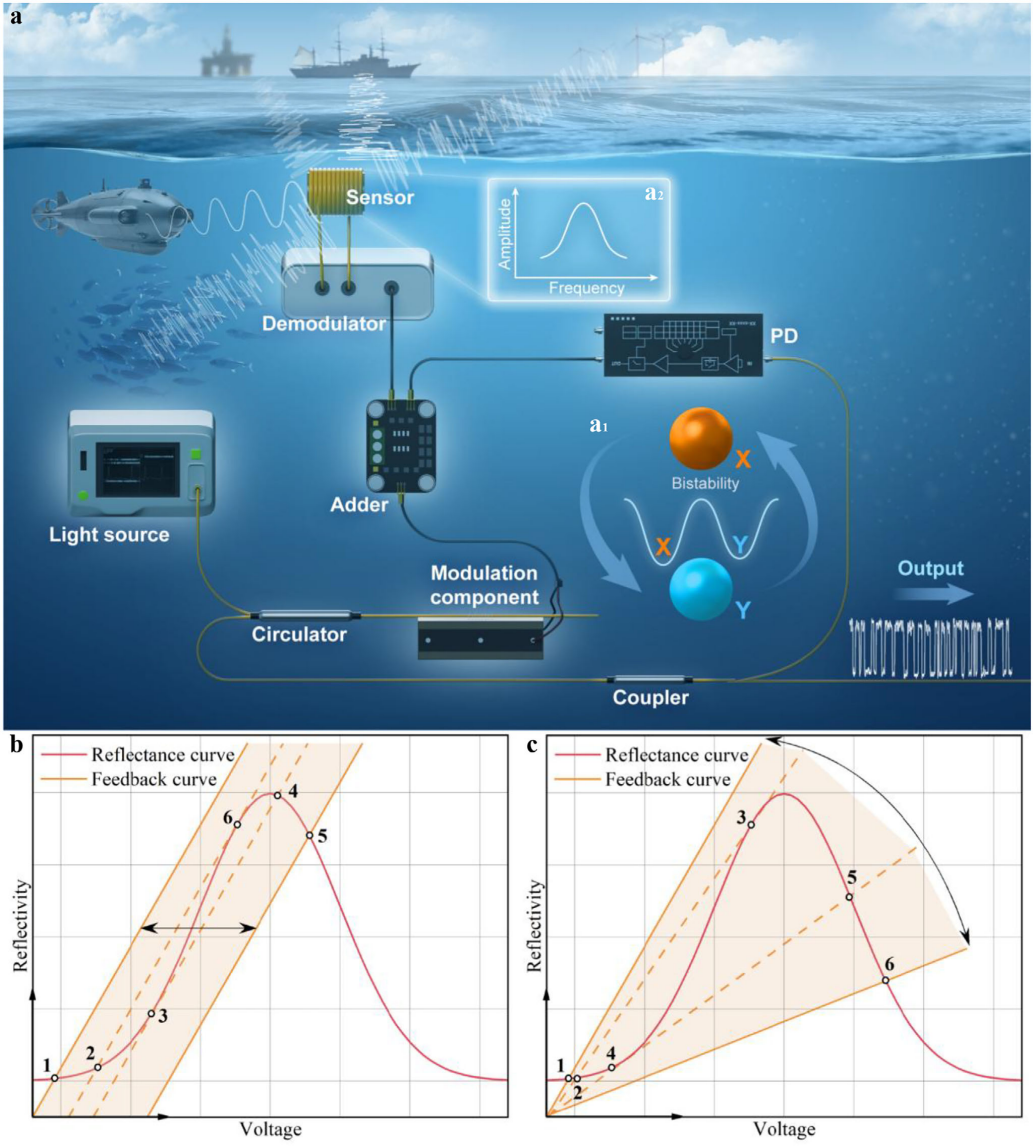
Principles of the MGSR sensing system. (a) Bistable output system based on the MGSR. Inset a_1_ illustrates the bistable transition effect generated within the negative feedback loop. Inset a_2_ shows the frequency response curve of the sensor at the resonant frequency. (b) Variation of the relative displacement between the feedback curve and the reflectivity curve with changes of the applied voltage. (c) Variation of the slope of the feedback curve with changes of the incident optical power.

Figure 2b,c illustrates the operational principle governing the bistable output. The FBG reflectivity exhibits a Gaussian profile as a function of input voltage due to the Bragg condition, characterizing the wavelength detuning process relative to the incident light power. Negative feedback from the FBG modulator stabilizes the system's operating point at the intersection of the reflectivity and feedback curves. By modulating the input voltage or optical power, these curves shift laterally, altering the intersection points. Consequently, a change in the positions of intersections induces an abrupt transition in the system output, thereby actualizing bistable switching. Theoretical derivations detailing how FBG feedback modulation achieves bistable output and ultimately MGSR output are provided in Note S3.

The FBG feedback platform provides a robust and reproducible framework for investigating optical bistability [62, 63, 64, 65]. Despite its established utility, the potential for harnessing this system for SR sensing remains untapped. To bridge this gap, we refined the system architecture and conducted an in‐depth investigation into the SR phenomena it generates. This approach establishes a versatile pathway for the broad application of all optical bistable systems in noise‐enhanced sensing. For further details on the fabrication and characterization of the bistable system, refer to the Method section.

### 2.4 Experimental Characterization of MGSR Sensing System

To experimentally substantiate MGSR, we established the bistable hardware architecture based on the analysis in Figure 2a, yielding the MGSR system shown in Figure 3a. We selected fiber‐optic hydrophones for this implementation, as their advances including inherent corrosion resistance, electromagnetic immunity, high sensitivity, and wide dynamic range render them ideally suited for rigorous underwater validation [66, 67, 68, 69]. It is worth noting that the implementation of closed‐loop feedback inevitably introduces signal delay, which may affect the matching condition of the Kramers escape rate. Therefore, we also investigated in depth the influence of this delay on the stochastic resonance effect, as detailed in Note S4.

**FIGURE 3:**
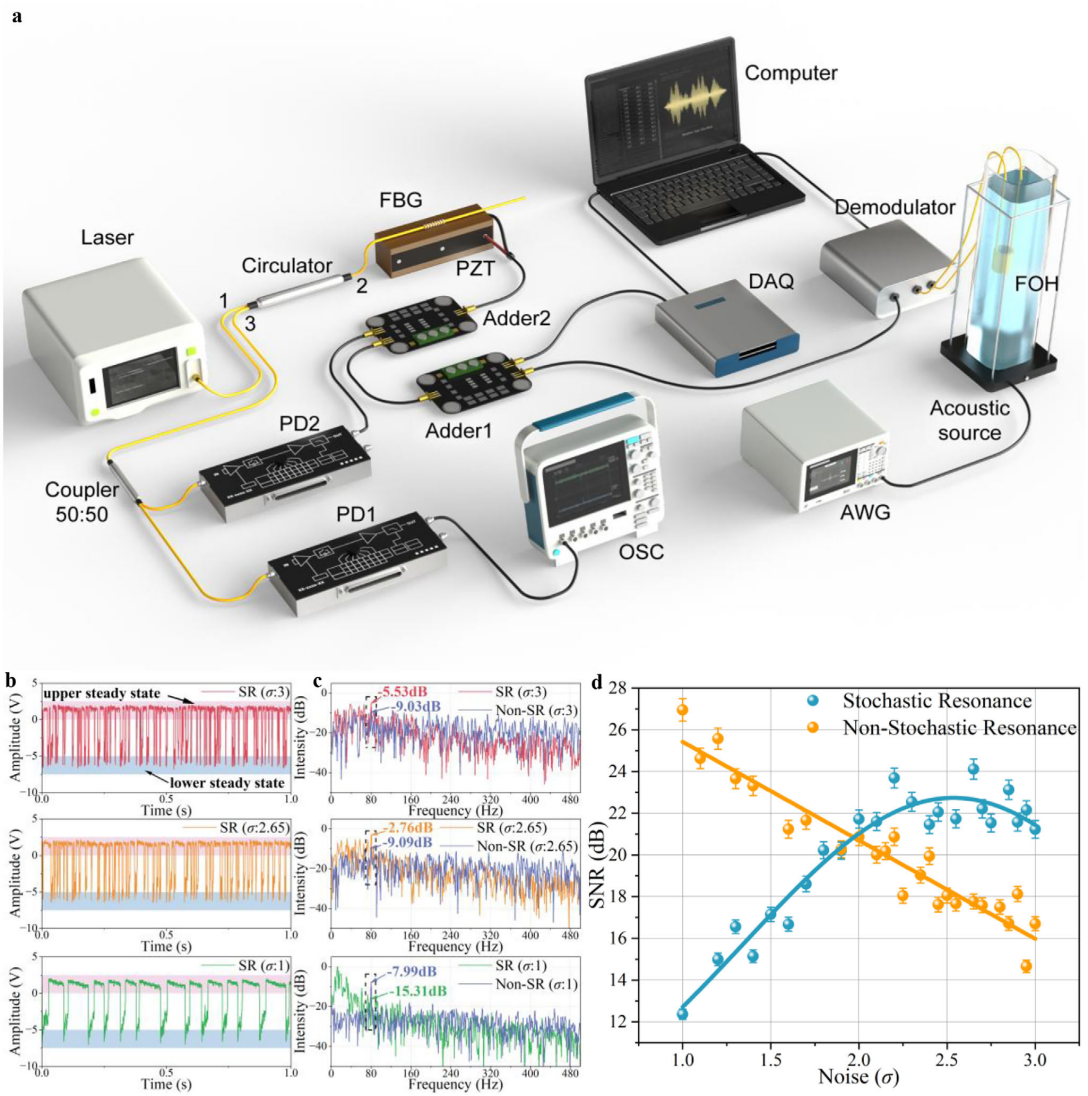
Verification of stochastic resonance effects of the MGSR sensing system. (a) The MGSR sensing system. In the figure, PZT denotes the piezoelectric ceramic, PD1 and PD2 are photodetectors, OSC represents the oscilloscope, AWG is the arbitrary waveform generator, DAQ stands for the data acquisition card, and FOH indicates the fiber‐optic hydrophone. (b) The time‐domain signal variations obtained after stochastic resonance processing under different noise intensity conditions. (c) Comparison of the frequency spectra obtained with and without stochastic resonance processing. (d) The variation curves of the SNR with and without stochastic resonance processing. *σ* stands for standard deviation.

To evaluate the system performance, we configured the input light source to a wavelength *λ* of 1549.95 nm and a power *P* of 3 mW. The signal generator was set to a frequency of 80 Hz with an amplitude of 100 mV, and these parameters were held constant. We then incrementally increased the standard deviation *σ* of the noise input to the data acquisition card while observing and recording the output on an oscilloscope. For clarity, the noise intensity *D* in the experiments is represented by the standard deviation *σ*, with *D* = *σ* ^2^ in the sense of characterizing the noise variance [70]. Throughout the following discussions, variations in the standard deviation σσ are therefore equivalent to variations in the noise intensity *D*. Figure 3b,c shows the resulting time‐domain and frequency‐domain signals obtained via stochastic resonance for *σ* values of 1, 2.65, and 3. As *σ* increases, the bistable waveforms in the time‐domain signals (Figure 3b) become progressively denser. Correspondingly, the 80 Hz signal peak in the frequency spectra (Figure 3c) first intensifies and then diminishes, its spectral intensity rises from an initial −15.31 dB to a maximum of −2.76 dB, before attenuating to −5.53 dB. In the absence of stochastic resonance, however, the 80 Hz peak intensity decreases monotonically with increasing *σ*, dropping from −7.99 dB to approximately −9 dB. These results demonstrate that the optimized bistable system can achieve stochastic resonance, thereby effectively enhancing a target signal buried in strong noise.

To quantify the specific enhancement effect of stochastic resonance on the target signal under strong background noise, we collated the frequency‐domain signals obtained under different noise standard deviations and calculated their SNRs. This allowed us to derive the SNR curves for conditions both with and without stochastic resonance. The processed results are presented in Figure 3d. As the noise standard deviation *σ* increases from 1.0 to 3.0 the SNR of the system without stochastic resonance processing exhibits a monotonic linear decrease, dropping from a maximum of 25.43 dB to a minimum of 15.98 dB. In contrast, under stochastic resonance conditions, the output SNR first increases and then decreases. This trend occurs because, with increasing noise intensity, the transition rate of the operating point successively approaches, satisfies, and ultimately deviates from the condition matching the Kramers rate to the driving signal frequency. Under this condition, a maximum SNR of 22.74 dB is achieved at a noise standard deviation of 2.54. Furthermore, compared to the case without stochastic resonance, the maximum SNR gain under identical noise conditions reaches 5.47 dB. This result essentially confirms the reliability of realizing stochastic resonance in the optimized bistable output system based on the fiber grating feedback mechanism. However, under the initial parameter settings (*λ* = 1549.95 nm, *P* = 3 mW), the observed SNR gain remains modest. Therefore, further investigation is required to elucidate the influence of system parameter variations on the effectiveness of stochastic resonance.

To investigate the influence of mechanical resonance on stochastic resonance, we varied the signal frequency from 90 to 180 Hz while maintaining a constant noise standard deviation of 3.0 and an input amplitude of 30 mV. Measurements were repeated three times per frequency, with the average SNR recorded as the output. As shown in Figure 4a, the SNR distribution follows a Gaussian profile, peaking at 23.88 dB for the 140 Hz resonant frequency. A comparison with the hydrophone's intrinsic frequency response, obtained following the procedure described in the Method section and shown in Figure S4c, reveals a strong correlation between the SNR and the mechanical resonance characteristics. This alignment directly substantiates the predictive validity of the MGSR theory.

**FIGURE 4:**
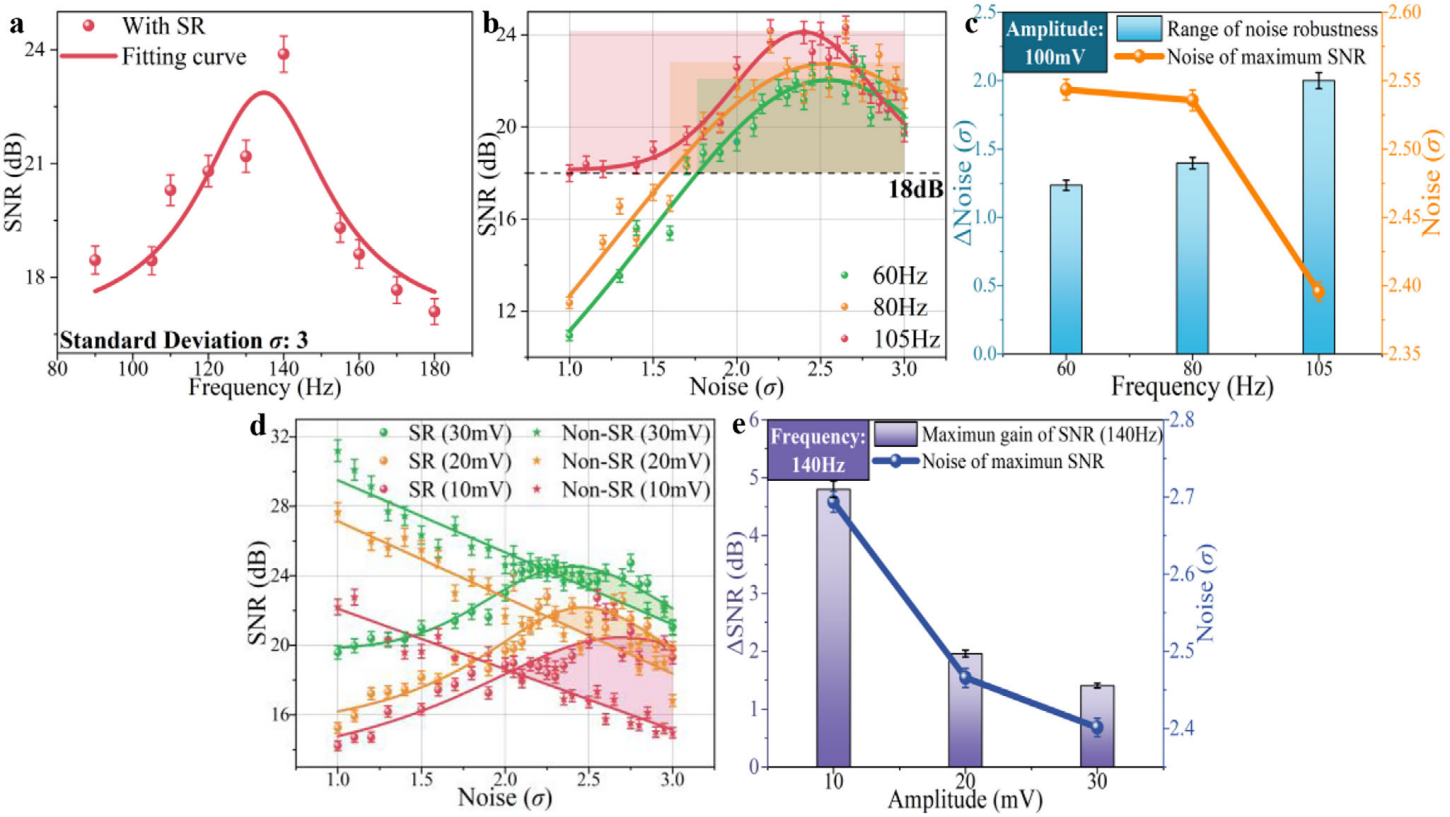
Validation of the MGSR sensing system for noise robustness, frequency selectivity, and weak signal gain effects. (a) SNR curves obtained from the stochastic resonance system as a function of signal frequency. (b) SNR curves obtained with stochastic resonance at different input signal frequencies. (c) Noise robustness and the noise intensity corresponding to the peak SNR in different frequencies. (d) Comparison of SNR curves with and without stochastic resonance processing at an input signal frequency of 140 Hz. (e) Noise intensity at the peak SNR and the peak SNR gain in different amplitude.

To gain further insight into the trend of the noise robustness as a function of frequency, and to specifically circumvent the interference from power‐line noise (e.g., at 50 Hz and 100 Hz), we selected test frequencies of 60 Hz and 105 Hz for this experiment. Building on our previous setup, the input signal amplitude from the generator was maintained at 100 mV, with the operational wavelength and optical power fixed at 1549.95 nm and 3 mW, respectively. By varying the noise intensity, we obtained the SNR curves both with stochastic resonance processing, and these results are presented in Figure 4b. The output SNR exhibits a characteristic as a function of frequency: when the input signal amplitude remains constant, the SNR increases monotonically with frequency under stochastic resonance conditions. When the threshold of the sensing system is set to 18 dB, the noise robustness range is shown as the marked region in Figure 4b. As the input signal frequency gradually approaches the resonant frequency, the noise robustness range improves. Specifically, as shown in Figure 4c, the noise standard deviation increases from 1.24 at 60 Hz to 2.0 at 105 Hz, and the enhancement covers almost the entire measurement range. Furthermore, by extracting the SNR curves under stochastic resonance conditions from Figure 4b, the optimal noise intensities corresponding to the SNR curves at various frequencies were compared, as illustrated in Figure 4c. The results reveal that as the frequency increases, the optimal noise intensity decreases from 2.54 to 2.40, exhibiting a systematic decreasing trend. These phenomena are attributed to the mechanical resonance characteristics of the MGSR. Specifically, when input signals near the resonant frequency undergo nonlinear gain, the height of the bistable potential barrier is reduced. Consequently, the optimal noise intensity required for stochastic resonance at the resonant frequency is lower than that at neighboring frequencies, indicating that signals near the resonant frequency are more readily excited into stochastic resonance compared to off‐resonant signals, which is consistent with Equation (13). In addition, because the mechanical resonance exhibits a Gaussian profile, the system suppresses the amplitude response at non‐resonant frequencies. All trends align with the behavior predicted by Figure 1e–h, which simultaneously reflect the noise robustness and frequency selectivity arising from the MGSR, thereby substantiating the reliability of the generalized Kramers escape rate theoretical model and the MGSR.

Notably, based on the optically tunable characteristics of the MGSR sensing system, we set the optical wavelength to 1549.90 nm and the optical power to 1.8 mW in subsequent experiments, thereby ensuring a positive gain in the stochastic resonance system. A detailed analysis of the mechanisms by which these adjustments influence the gain effect is provided in Note S5.

Although we have previously conducted a detailed investigation of the effect of frequency variation on MGSR, the influence of varying the input signal amplitude at a fixed frequency remains unexplored. Given that amplitude variations are most pronounced at the resonant frequency, we selected 140 Hz as the carrier frequency for subsequent amplitude‐dependent testing. By systematically reducing the input amplitude from 30 to 10 mV at the signal generator while keeping all other parameters constant, we acquired and processed the SNR curves for both stochastic resonance‐processed and unprocessed states, and the results are shown in Figure 4d. As the input amplitude decreases, the SNR for both conditions exhibits a general downward trend. However, the gain interval introduced by MGSR progressively widens with signal attenuation. Figure 4e reveals that as the amplitude declines from 30 to 10 mV, the peak gain of the fitted SNR curve obtained via MGSR increases from 1.40 to 4.80 dB. Concurrently, the noise intensity required to achieve maximum SNR also varies with amplitude, with the optimal noise standard deviation rising from 2.40 to 2.69. This behavior is consistent with Equation (13), which predicts that a decrease in input amplitude leads to an increase in the required optimal noise intensity. The shift toward higher noise levels enables the MGSR processing of a 10 mV input signal to yield greater gain under a strong noise background than that achievable with a 30 mV input. These results demonstrate that MGSR exhibits a higher efficacy in extracting weaker signals, as the reduced input amplitude necessitates a higher noise threshold to activate the nonlinear bistable transition.

Thus far, by varying the amplitude and frequency of the input signal, we have verified the reliability and feasibility of the MGSR. Concurrently, we have demonstrated that adjusting the optical wavelength or power can modulate the residence time fraction of the operating point at different steady states, or alter the external voltage required to induce stochastic resonance (see Note S5 for details). Building on this sensing scheme, the reported system holds significant promise for achieving an even higher SNR gain.

### 2.5 Application of MGSR Sensing System with Realistic Ocean Ambient Noise

To assess the practical applicability of the MGSR system in realistic oceanic environments, we replaced Gaussian white noise with recorded ocean ambient noise. Unlike Gaussian white noise, which exhibits a constant power spectral density (PSD), ocean ambient noise is characterized by energy predominantly concentrated in the very‐low‐frequency band, with its PSD gradually decreasing as frequency increases [71]. Analysis of Figure 1a suggests that the complex composition of ocean ambient noise renders pure computer simulations insufficient for fully replicating its effects. Accordingly, we recorded three 640 s ambient noise segments at different times in the Xiamen sea area, China. The measurement setup and the three time‐domain noise signals, denoted as Noise 1, Noise 2, and Noise 3, are presented in Figure 5a. Figure 5b shows the corresponding PSD profiles, which consistently exhibit a decreasing trend with increasing frequency, confirming that the primary noise energy is concentrated near very low frequencies. This observation aligns with the analysis above.

**FIGURE 5:**
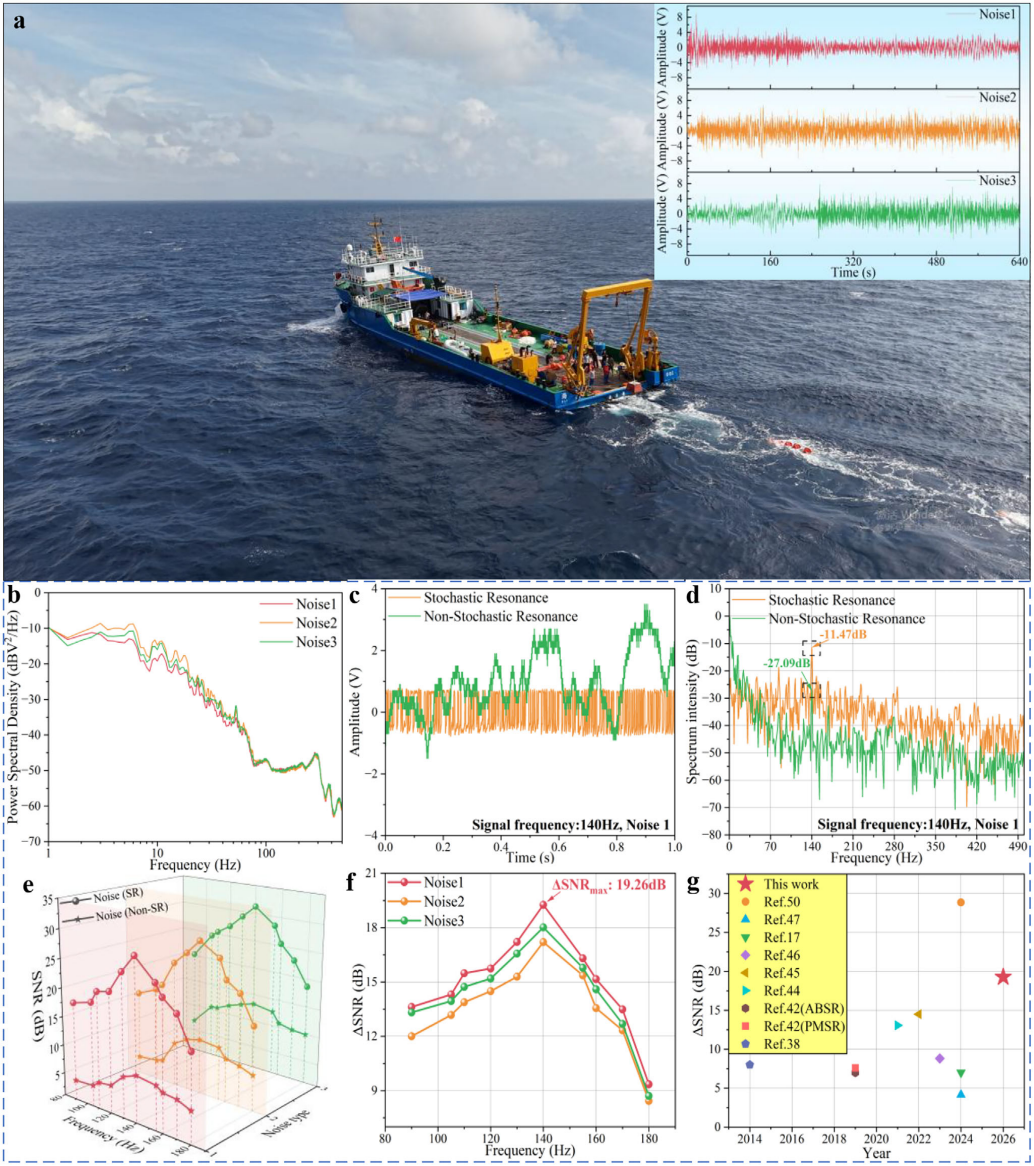
Performance of the MGSR sensing system under ocean ambient noise. (a) The ocean noise environment (Xiamen, China) and the time‐domain signals of ocean ambient noise collected at different times. (b) The variation in the power spectral density of the ocean ambient noise at different times. (c) Comparison of the output time‐domain signal processed by stochastic resonance versus the output time‐domain signal without stochastic resonance processing. (d) Comparison of the output frequency‐domain signal processed by stochastic resonance versus the output frequency‐domain signal without stochastic resonance processing. (e) The SNR curves as a function of frequency for the stochastic resonance and non‐stochastic resonance systems under the influence of different ocean ambient noises. (f) The gain variation curves of the stochastic resonance system compared to the non‐stochastic resonance system under different ocean ambient noise conditions. (g) Comparison of SNR gain with previous studies on stochastic‐resonance‐based acoustic sensing.

When the noise source was switched from Gaussian white noise to ocean ambient noise, the MGSR system configuration shown in Figure 3a remained unchanged, while the optical wavelength and power were adjusted based on the analysis in Figure S7a–d. It should be noted that the workflow for optimizing and selecting system parameters in practical applications of the MGSR sensing system can be found in Note S6. Specifically, the wavelength was maintained at 1549.90 nm and the power was set to 1.08 mW. To preliminarily evaluate the MGSR effect under ocean ambient noise, Noise 1 was used as the input noise with an external signal amplitude of 1 mV, and the results are presented in Figure 5c,d. In Figure 5c, owing to the concentration of ocean ambient noise energy in the very‐low‐frequency range, only a sparse time‐domain waveform was obtained without MGSR processing. However, after applying MGSR to the input signal, the low‐frequency noise energy was redistributed to frequencies near the target signal, resulting in a bistable waveform in the time domain. Examining the frequency spectrum after stochastic resonance processing in Figure 5d, the spectral peak at 140 Hz increased from −27.09 to −11.47 dB, providing preliminary evidence that the proposed MGSR system enhances the output of weak external signals under ocean ambient noise conditions.

To thoroughly investigate the SNR improvement achieved by MGSR under ocean background noise, we swept the target signal frequency from 90 to 180 Hz while maintaining a constant amplitude, and the system output SNR was measured with and without stochastic resonance. At each frequency point, measurements were quintuplicated and averaged, yielding SNR curves under three noise sample sets, as shown in Figure 5e. Compared to the case without stochastic resonance, the SNR after MGSR processing is significantly higher, given the external signal amplitude of only 1 mV. Based on the results in Figure 5e, we further compiled them into the SNR gain curves obtained under different noise samples. As shown in Figure 5f, when the MGSR sensing system operates within the noise range exhibited by Noise 1 to Noise 3, the SNR gain at the mechanical resonance frequency ranges from 17.20 to 19.26 dB, representing a 13.79 dB maximum enhancement over the SNR gain observed in Figure 3d. These results indicate that, in the context of ocean ambient noise, the MGSR gain can be further enhanced through appropriate optical wavelength, power settings, and the use of the MGSR. As shown in Figure 5g, compared with previous literature employing stochastic resonance schemes in acoustic sensing [17, 40, 44, 46, 47, 48, 49], our system achieves a higher SNR gain than previous research and also exceeds current state‐of‐the‐art acoustic sensors that implement stochastic resonance via hardware, highlighting the performance advantages of the MGSR. It should be noted that although some existing schemes, such as DSRA [52], achieve a higher gain than our reported work, the DSRA scheme is based on offline digital post‐processing. In contrast, the MGSR sensing system exhibits better real‐time signal processing performance. Therefore, the advantages of the MGSR sensing system remain irreplaceable.

## 3 Discussion

In conclusion, by using an optimized bistable system enhanced by a fiber Bragg grating feedback mechanism, we experimentally demonstrated MGSR at the hardware level in underwater acoustics. This approach not only achieves gain for weak signals but also enables targeted enhancement of stochastic resonance processing for specific frequencies while suppressing outputs at other frequencies, thus offering frequency selectivity and high noise robustness. In addition, unlike previous approaches that achieved stochastic resonance through offline digital post‐processing or electronic hardware, the MGSR sensing system primarily uses optical fiber for signal transmission and features an all‐optical sensing front end. This design offers several advantages, including high response bandwidth, large dynamic range, corrosion resistance, and immunity to electromagnetic interference. Moreover, the MGSR sensing system avoids the poor real‐time performance inherent in post‐processing while also reducing fabrication difficulty, thereby lowering the barrier to deploying stochastic resonance sensing technology in practical underwater environments. Notably, preliminary experimental results suggest that the system's output gain can be further improved and stabilized through optimization strategies such as introducing adaptive mechanisms to dynamically adjust optical wavelength or power. These adjustments can modulate the dwell time in a single steady state or the threshold voltage for state switching, thereby dynamically adjusting the initial position of the bistable potential barrier and enabling the system to adapt to varying ocean ambient noise conditions. Furthermore, the structural decoupling of the sensing and processing units not only grants this scheme considerable versatility, enabling straightforward adaptation to diverse acoustic or vibration sensors by simply modifying the structure of the sensing end, but also allows for the independent optimization of the interrogation electronics, which lays a foundation for further improving the MGSR sensing system in subsequent work. Consequently, this work thereby establishes a versatile platform for real‐time, high‐fidelity sensing in applications ranging from pipeline monitoring and seismic early warning to gravitational wave detection, underscoring its broad technological impact (see Note S7 for details).

It is worth noting that, in future work, beyond the optical parameter adaptive modulation discussed above, the MGSR sensing system still deserves further investigation in the following aspects. The first is the thermal noise floor introduced by electronic components (see Materials and methods section). When the environmental noise intensity is insufficient, this noise floor can serve as a beneficial supplement, driving the bistable system toward the optimal stochastic resonance state. However, when the environmental noise fluctuates violently, the fixed thermal noise floor may hinder the system from achieving optimal gain. To maintain the stability of the system gain, in addition to adaptively modulating the optical power and wavelength, inspired by the work of Guo et al., the integration of back‐end digital lock‐in amplification technology offers a potential pathway for future optimization of the MGSR sensing system. By enabling narrowband extraction of target signals from high‐intensity background noise, lock‐in amplification is expected to extend the dynamic range of the system with respect to external noise input [72].

Meanwhile, although the frequency selectivity of the current MGSR sensing system facilitates the enhancement of individual target line spectra, it may introduce limitations when processing broadband signals such as ship‐radiated noise. Broadband signals contain multiple line‐spectrum components spanning a wide frequency range, whereas the mechanical resonance of the sensor preferentially amplifies signals near the resonant frequency while suppressing off‐resonance components. Improper handling may distort the spectrum of the target signal fed into the MGSR sensing system, thereby affecting subsequent signal processing. A practical strategy to mitigate this limitation is to employ an array of sensors with different mechanical resonant frequencies. Before the broadband signal is fed into the bistable feedback loop, the mechanical resonance effects of the array can extract multiple specific frequency components of interest, enabling the system to selectively and simultaneously enhance multiple target line spectra, thus improving the capability of the MGSR sensing system in extracting broadband signals. Furthermore, given that multiple line‐spectrum signals may need to be processed simultaneously within the system, future efforts should also optimize the multi‐frequency synchronous processing capability of the MGSR sensing system. For example, employing an FBG array by distinguishing the inherent properties of individual FBGs, including their initial reflection central wavelengths and reflectivities, combined with the coordinated modulation of optical power and wavelength, would allow the FBG array to process multiple line‐spectrum signals concurrently, ultimately achieving gain for broadband signals.

In addition, reducing the deployment cost and spatial footprint of the MGSR sensing system is also a critical consideration for future large‐scale applications. A detailed discussion of this aspect can be found in Note S8. Overall, by advancing stochastic resonance from algorithmic post‐processing to hardware‐level real‐time implementation, the MGSR sensing system offers an all‐optical solution for underwater weak‐signal detection that simultaneously provides frequency selectivity, noise robustness and immunity to electromagnetic interference. This work not only validates the physical mechanism of mechanically‐gated stochastic resonance at the fundamental level, but also lays a technical foundation for subsequent system optimization in terms of integration, multi‐band expansion and engineering deployment.

## 4 Materials and Methods

### 4.1 FBG Reflectivity Curve Testing

To demonstrate the output characterization of the bistable system, we employed a preliminary experimental setup based on a fiber Bragg grating bonded to a PZT actuator with an initial central wavelength of 1550.02 nm and a reflectivity of 0.61. The setup to measure the reflectance of the fiber Bragg grating is illustrated in Figure S2a. Specifically, the fiber Bragg grating is affixed to the surface of the PZT using UV adhesive, ensuring that the PZT can strain the grating upon deformation. To obtain the reflectance variation curve, the output wavelength *λ* and optical power *P* of the light source were set to 1550.06 nm and 8 mW, respectively. A DC power supply providing voltage *V_d_* was applied to the PZT to modulate the period *Λ* of the fiber grating, thereby shifting the Bragg central wavelength. The relationship between the Bragg central wavelength and the grating period is expressed as [73]:

(16) $$\lambda_{B}=2n_{eff}\Lambda$$

Here, *n_eff_* represents the effective refractive index of the fiber core. Under the condition that the output wavelength and optical power of the light source remain constant, the outputs from channels 2 and 3 of the circulator were measured using optical power meters, which allows the determination of the relationship between the grating reflectivity and the input voltage, as shown in Figure S2b. The normalized reflectivity curve exhibits an overall Gaussian profile, which is consistent with the reflectivity distribution shown in Figure 2a,b in the main text. The reflectivity reaches its maximum when a DC voltage of 5 V is applied, thereby laying the foundational principle for the subsequent experiments to output bistable signals via the fiber Bragg grating feedback mechanism.

However, external temperature variations may induce a slight drift in the effective refractive index of the fiber core *n_eff_*, As indicated by Equation (16), this drift alters the initial Bragg wavelength of the grating. Therefore, prior to each measurement, it is essential to ensure the reflectance spectrum of the fiber Bragg grating exhibits an expected Gaussian distribution by adjusting the input optical power and wavelength accordingly. More detailed control strategies for the thermal drift of the fiber Bragg grating can be found in Note S9.

### 4.2 Bistable Curve Measurement

Following the acquisition of the reflectance spectrum, we implemented the feedback architecture depicted in Figure S3a. Maintaining the optical wavelength and power at 1550.06 nm and 8 mW, respectively, a DC voltage *V_d_* was swept from −10 to +10 V in 0.5 V increments. The resulting optical hysteresis curve is presented in Figure S3b. As the DC voltage increased from −10 V to +10 V, the signal peak exhibited a state transition at 1 V. Conversely, during the downward sweep from +10 to −10 V, the state transition occurred at −7 V. This behavior indicates that the fiber Bragg grating feedback structure exhibits a hysteresis window between −7 and 1 V (shaded in blue in Figure S3b). Defining the voltage interval from −10 to 1 V (starting from −10 V) as the lower steady state, and the interval from +10 V to −7 V (starting from +10 V) as the upper steady state, mutual transitions between these two steady states occur when the input voltage variation of the acquisition card exceeds 8 V. Thus, replacing the DC voltage with a time‐varying voltage signal enables the operating point to continuously switch between the two steady states, ultimately yielding a bistable signal output. Notably, analysis of the operating principle of the bistable system in Figure 2 indicates that the width of the hysteresis window is governed by the initial optical power and wavelength. This tunability provides a vital technical foundation for actively adapting stochastic resonance systems to dynamic noise environments.

### 4.3 Experimental Setup of the MGSR System

The structure of the MGSR sensing system is illustrated in Figure 3a. The system employs a tunable laser source (Santec, TSL‐570), which facilitates convenient adjustment of the optical power and wavelength. Additionally, we fabricated a push‐pull structured fiber‐optic hydrophone by winding a Michelson interferometer around a resin sleeve, as detailed in Figure S4a. The external voltage signal primarily consists of signals from a data acquisition (DAQ, NI USB‐6353) card and the output signal from a demodulator. The DAQ card signal is used to generate Gaussian white noise, simulating the noise interference encountered by the sensor in underwater acoustic sensing while the demodulator functioned dually as a secondary light source and as a processor for interferometric signals. Finally, the demodulated phase signal is converted into voltage, mixed with the aforementioned DAQ card signal via an adder (OPA1678) and routed to the PZT, which adjusts the central Bragg wavelength of the fiber grating to achieve a bistable output. It should be noted that the demodulator primarily employs a PGC (Phase Generation Carrier) modulation and demodulation scheme to process the acquired raw signals, as detailed in Note S10.

Notably, these electronic feedback components (e.g., photodetectors and adders) introduce an additional thermal noise floor. However, within the stochastic resonance framework, the performance of the MGSR system is not fundamentally limited by this noise floor. Rather, it can synergistically combine with external environmental noise and interact with the target signal and the bistable potential barrier, thereby essentially acting as an additional noise source that contributes to satisfying the Kramers escape rate matching condition (*r_k_* = 2*f*). Therefore, provided that the total noise intensity remains within the theoretically predicted optimal noise intensity range, it can equally participate in the stochastic resonance process, rather than merely degrading the SNR.

### 4.4 Mechanical Resonance Characterization of the Hydrophone

The fiber‐optic hydrophone was fabricated as previously described. To enable MGSR validation, we characterized its mechanical resonance by measuring the frequency response within a standing wave tube, as depicted in Figure S4b. In this configuration, the demodulator incorporates a built‐in light source, and an attenuator is employed to modulate the optical signal intensity, preventing excessively strong interference signals that could damage the demodulator. The fiber‐optic hydrophone under test was placed inside a standing wave tube. The amplitude of the input acoustic signal was maintained constant at 1 V, while its frequency was incrementally adjusted from 20 to 180 Hz. Finally, a computer was used to acquire and process the demodulated signal data, yielding the output variation of the tested fiber‐optic hydrophone as a function of frequency. As shown in Figure S4c, the frequency response curve exhibits an overall Gaussian profile between 20 and 180 Hz, with the demodulated phase value reaching a peak of 417.40 rad at a frequency of 140 Hz, which we attribute to the radial breathing mode of the elastic tubes (see Note S11) and identify as the mechanical resonance frequency, providing the essential experimental benchmark for stochastic resonance parameter optimization.

## Author Contributions

**Xuyu Zhang**: conceptualization, investigation, writing – original draft, methodology, validation, formal analysis, data curation, visualization. **Minzheng Sun**: writing – review and editing, resources, data curation, investigation, methodology, software. **Cunzheng Fan**: writing – review and editing, investigation, formal analysis, methodology. **Shuolong Zhu**: investigation, writing – review and editing, formal analysis, methodology. **Qilin Hong**: supervision, resources, writing – review and editing, conceptualization. **Pan Xu**: funding acquisition, resources, data curation, writing – review and editing. **Min Zhu**: funding acquisition, writing – review and editing, supervision, resources. **Zhengliang Hu**: funding acquisition, supervision, writing – review and editing, resources. **Yongchao Zou**: supervision, writing – review and editing, methodology, conceptualization, investigation, formal analysis, resources. **Qizhen Sun**: writing – review and editing, methodology, conceptualization, supervision, investigation, resources, formal analysis.

## Conflicts of Interest

The authors declare no conflicts of interest.

## Supporting information




**Supporting File**: advs78634‐sup‐0001‐SuppMat.docx.

## Data Availability

The data that support the findings of this study are available from the corresponding author upon reasonable request.
