# Supplementary material for: Mechanically‐Gated Stochastic Resonance for All‐Optical Acoustic Sensing in Noisy Environments
Source: Adv Sci (Weinh). 2026 Nov 5:e78634. Online ahead of print. doi: 10.1002/advs.78634 (PMC13735441; doi:10.1002/advs.78634)
Supplement: Supplementary file 1 [file ADVS-9999-e78634-s001.docx]

Supplementary Information for

**Mechanically-Gated Stochastic Resonance for All-Optical Acoustic Sensing in Noisy Environments**

Xuyu Zhang^1^, Minzheng Sun^1^, Cunzheng Fan^2^, Shuolong Zhu^2^, Qilin Hong^1^, Pan Xu^1^, Min Zhu^1^, Zhengliang Hu^1^, Yongchao Zou^1*^, and Qizhen Sun^2*^

^1^College of Meteorology and Oceanography, National University of Defense Technology, Changsha, Hunan, 410073, China

^2^National Engineering Research Center for Next Generation Internet Access System (NGIA), School of Optical and Electronic Information, Huazhong University of Science and Technology, Wuhan, Hubei, 430074, China.

*Corresponding authors: Yongchao Zou and Qizhen Sun

E-mails: [zouyongchao@nudt.edu.cn](mailto:*zouyongchao@nudt.edu.cn) and [qzsun@mail.hust.edu.cn](mailto:qzsun@mail.hust.edu.cn,)

**This PDF file includes:**

Supplementary Notes 1 to 11

Supplementary Figs. 1 to 8

Supplementary Tables 1 to 2

References (1 to 20)

**Supplementary Note 1. Derivation of the MGSR theory under acousto-structural coupling**

In the analysis of the main text, we have basically established the theoretical framework of MGSR, but its practical application in the field of underwater acoustic sensing has not yet been fully explored. To this end, we need to start from the process of acquiring acoustic signals through acousto-structural coupling and gradually derive the MGSR theory applicable to practical environments. Fig. 3(a) of the main text shows the structure of the MGSR sensing system, in which the external acoustic signal is acquired through demodulation of the hydrophone by a demodulator, and we define *V_ac_*(*t*) as the demodulated voltage from the hydrophone, and *A_eff_*(*f*) is the effective driving amplitude obtained from the acoustic pressure via the hydrophone transfer function *H_h_*(*f*). The acousto-structural coupling that converts the acoustic pressure into the demodulated voltage is derived in Supplementary Note 11.

In the preceding discussion, *A_c_* represents the amplitude of the input signal at which the potential barrier of the bistable system completely vanishes. We adopt the time-harmonic convention for the acoustic field^1^:

$p(r,t)=\Re\left[\tilde{p}(r)e^{i\cdot2\pi ft}\right]$ (S1)

where *p* is the acoustic pressure, $\tilde{p}(r)$ is its complex amplitude, *r* is the spatial position vector, *t* is time, *f* is the signal frequency, and *ℜ*[·] denotes the real part. The acoustic pressure $\tilde{p}(r)$ is then converted into the demodulated voltage of the hydrophone through the acousto-structural coupling transfer function *H_h_*(*f*) (see Supplementary Note 11). The resulting demodulated voltage is:

 (S2)

Here, *ϕ* represents the total initial phase variation caused by factors such as acousto-structural coupling and time delays generated during the propagation of external signals from the sound source to the hydrophone. The effective driving amplitude *A_eff_*(*f*) is influenced by the resonant response of the hydrophone, and this variable can be further decomposed as:

$A_{eff}(f)=\left|H_{h}(f)\right|\cdot\left|\tilde{p}(r)\right|$ (S3)

where *f* is the signal frequency. Within the framework of the bistable potential, this demodulated voltage serves as the periodic driving term in the Langevin equation:

$S(t)\equiv V_{ac}(t)$ (S4)

Substituting the bistable potential *U*(*x*) into Eq. (1) and neglecting the effect of the noise signal yields:

$\frac{dx}{dt}=ax-bx^{3}+S(t)$ (S5)

where *x* is the system operating point position, and *a* and *b* are the bistable system constants defined in Eq. (2) of the main text. The periodic driving acts as an instantaneous tilt on the bistable potential. The instantaneous potential is^2^:

$U(x,t)=-\frac{a}{2}x^{2}+\frac{b}{4}x^{4}-S(t)\cdot x$ (S6)

The corresponding instantaneous steady-state equation is:

$bx^{3}-ax-S(t)=0$ (S7)

Eq. (S7) is a cubic equation in *x*, with *S*(*t*) acting as an instantaneous tilt. Its discriminant is:

$\Delta(t)=4a^{3}b-27b^{2}S^{2}(t)$ (S8)

When Δ(*t*) > 0, Eq. (S7) has three distinct real roots, corresponding to the potential barrier and two potential wells. When Δ(*t*) < 0, Eq. (S7) has only one real root and two complex conjugate roots, and the system transitions to a monostable state. The barrier vanishes when Δ(t) = 0, which corresponds to the instant when the instantaneous amplitude |*S*(*t*)| reaches the critical value *A_c_*. The critical amplitude is^3^:

$A_{c}=\sqrt{\frac{4a^{3}}{27b}}$ (S9)

The time-dependent barrier height is obtained as:

$\Delta U(t)=\Delta U_{0}\cdot\left\{1-\left[\frac{S(t)}{A_{c}}\right]^{2}\right\}^{\frac{3}{2}}$ (S10)

where Δ*U*_0_ is the static barrier height. When *S*(*t*)=0, the barrier height reduces to Δ*U*_0_ = *a*^2^/(4*b*) (see Eq. (3) in the main text), and vanishes when |*S*(*t*)| = *A_c_*. From the analysis in the main text, the occurrence of bistable stochastic resonance mainly depends on the moment when the barrier is lowest, that is, when |*S*(*t*)| is maximum. Therefore, we primarily adopt the maximum amplitude *A_eff_*(*f*) for subsequent derivations. Thus, by replacing *S*(*t*) with *A_eff_*(*f*), we obtain the bistable potential function at a given signal frequency *f* as:

$U(x)=-\frac{a}{2}x^{2}+\frac{b}{4}x^{4}-A_{eff}(f)\cdot x$ (S11)

The corresponding steady-state equation is given by:

$bx^{3}-ax-A_{eff}(f)=0$ (S12)

When the discriminant Δ > 0, that is, |*A_eff_*(*f*)| < *A_c_*, Eq. (S12) has three distinct real roots *x*_1_[*A_eff_*(*f*)] < *x*_2_[*A_eff_*(*f*)] < *x*_3_[*A_eff_*(*f*)], where *x*_1_[*A_eff_*(*f*)] and *x*_3_[*A_eff_*(*f*)] correspond to the potential wells and *x*_2_[*A_eff_*(*f*)] to the barrier. Using the trigonometric solution of the cubic equation, the roots are:

$x_{k}\left[A_{eff}(f)\right]=2\sqrt{\frac{a}{3b}}\cos\left\{\frac{1}{3}\arccos\left[\frac{A_{eff}(f)}{A_{c}}\right]-\frac{2\pi k}{3}\right\},k=0,1,2$ (S13)

Meanwhile, the barrier height can be expressed as:

$\Delta U\left[A_{eff}(f)\right]=U(x_{2})-U(x_{1})=\Delta U_{0}\cdot\left\{1-\left[\frac{A_{eff}(f)}{A_{c}}\right]^{2}\right\}^{\frac{3}{2}}$ (S14)

This expression is an analytical result of the tilted bistable potential. It reduces to Δ*U*_0_ at *A_eff_*(*f*) = 0 and vanishes at *A_eff_*(*f*) = *A_c_*. Finally, we obtain the Kramers escape rate based on the hydrophone resonant response and its corresponding optimal noise, respectively given by^4^^,^ ^5^:

$\left\{\begin{matrix} r_{k}\left[A_{eff}(f)\right]=\frac{1}{2\pi}\sqrt{U''\left\{x_{1}\left[A_{eff}(f)\right]\right\}\left|U''\left\{x_{2}\left[A_{eff}(f)\right]\right\}\right|}\exp\left\{-\frac{\Delta U\left[A_{eff}(f)\right]}{D}\right\} \\ D_{opt}(f)=\frac{\Delta U\left[A_{eff}(f)\right]}{\ln\left\{\frac{\sqrt{U''\left\{x_{1}\left[A_{eff}(f)\right]\right\}\left|U''\left\{x_{2}\left[A_{eff}(f)\right]\right\}\right|}}{4\pi f}\right\}} \end{matrix}\right.$ (S15)

where *D* is the noise intensity defined in Eq. (2) of the main text. The curvature term $U''(x)$ denotes the second derivative of the bistable potential, given by:

$U''(x)=-a+3bx^{2}$ (S16)

When *A_eff_*(*f*) = 0, Eq. (S15) yields the positions of one potential well and the barrier as $x_{1}=-\sqrt{a/b}$ and x_2_ = 0, respectively. Substituting these into the curvature term gives $U''(x_{1})=2a$ and $\left|U''(x_{2})\right|=a$. The Kramers rate then recovers the classical result^4^:

$r_{k}=\frac{\sqrt{2}a}{2\pi}\exp\left(-\frac{a^{2}}{4bD}\right)$ (S17)

It is worth examining the upper limit of the escape rate implied by Eq. (S15). As *D* → ∞, the exponential term approaches unity, and the escape rate limits at:

$r_{kmax}\left[A_{eff}(f)\right]=\frac{1}{2\pi}\sqrt{U''\left\{x_{1}\left[A_{eff}(f)\right]\right\}\left|U''\left\{x_{2}\left[A_{eff}(f)\right]\right\}\right|}$ (S18)

In the static limit *A_eff_*(*f*) = 0, Eq. (S18) recovers the classical result $r_{kmax}=a/\left(\sqrt{2}\pi\right)$. As *A_eff_*(*f*) increases toward *A_c_*, the barrier top becomes progressively flattened, so that and *r_kmax_*[*A_eff_*(*f*)] decreases. This behavior is a direct consequence of the mechanical resonance. The mechanical resonance substantially reduces the barrier height Δ*U*[*A_eff_*(*f*)], so that the resonance condition *r_k_* = 2*f* can be satisfied with a much smaller, physically meaningful noise intensity *D*. This is the physical origin of the dip in the optimal noise curve near the resonant frequency, as shown in Fig. 1(d) of the main text.

Through these derivations, we have essentially established the theoretical framework of MGSR in the field of underwater acoustic sensing. However, its implementation in practical physical systems still requires the integration of a fiber Bragg grating feedback mechanism. The detailed theoretical derivation of this aspect can be found in Supplementary Note 3.

**Supplementary Note 2. Verification of the generalized Kramers escape rate theoretical model**

To validate the efficacy of the theoretical model in predicting the optimal noise intensity, we established a bistable stochastic resonance system model. This was done under the premise of introducing a standard normal distribution scaled by standard normal distributions with a mean of 0 and a variance of 1. We investigated the variation in optimal noise intensity under two conditions: without mechanical resonance and with mechanical resonance. In the absence of mechanical resonance, the signal amplitude $\tilde{A}_{0}$is taken as 0.465. When the input signal frequency increases from 0.1 Hz to 0.8 Hz, the resulting optimal noise intensity curve is shown in Supplementary Fig. 5(a). For input signal frequencies of 0.3, 0.4, 0.5, 0.6, and 0.7 Hz, the SNR curves obtained from the bistable stochastic resonance system model are presented in Supplementary Fig. 5(b). It can be observed that, compared to the original Kramers' escape rate theoretical model, which only considers the influence of signal frequency on the optimal noise intensity curve, the generalized Kramers' escape rate theoretical model incorporates the signal amplitude as a factor affecting the optimal noise intensity, alongside the signal frequency. Consequently, its overall predictive performance is closer to the peak SNR output derived from stochastic resonance processing. Based on this, by obtaining the actual optimal noise intensities at different frequencies, it is evident that the relative prediction error of the generalized model results is 3.1%, which is substantially lower than the 22.9% relative error of the original model results, as shown in Supplementary Fig. 5(c). Considering that the stochastic resonance phenomenon is only statistically significant and the deterministic relationship between input and output cannot be precisely obtained^4^, a relative prediction error controlled within 5% is considered ideal.

When considering the effects of mechanical resonance, the effective amplitude of the input signal exhibits a Gaussian dependence on frequency. After introducing a mechanical resonance response with a Q-factor of 2.12 (inset of Supplementary Fig. 5) and a signal peak value of 0.697, the generalized theoretical model indicates that, compared with the optimal noise intensity derived from the original Kramers escape rate theory, the optimal noise intensity decreases near the resonant frequency, as shown in Supplementary Fig. 5(d). After sequentially inputting signal frequencies of 0.3, 0.4, 0.5, 0.6, and 0.7 Hz into the bistable stochastic resonance system model, the resulting SNR curves are presented in Supplementary Fig. 5(e). In conjunction with the aforementioned prediction of optimal noise intensity, it is evident that the generalized theoretical model is also applicable to stochastic resonance outputs influenced by mechanical resonance effects. The relative prediction error is 7.0% (refer to Supplementary Fig. 5(f)), which is significantly better than the 30.2% relative prediction error of the original model results. It should be noted that although the relative prediction error of the generalized theoretical model under resonant conditions is higher than that under non-resonant conditions, considering that the input signal amplitude in the former case varies nonlinearly with frequency, and combined with the earlier judgment that stochastic resonance phenomena are only statistically significant, a relative error of 7.0% is acceptable.

In summary, by generalizing Kramers' escape rate theory, we have effectively predicted the variations in stochastic resonance output under resonant driving, thereby providing a more accurate theoretical tool for subsequent research.

**Supplementary Note 3. Theory of bistable output based on fiber Bragg grating feedback mechanism**

In the main text, we have clarified that modulating the input voltage and input optical power can shift the operating point of the system, thereby enabling bistable signal output. Fig. 2(b) illustrates the operating principle of the proposed voltage-based switching scheme. Assuming the initial operating point is at Position 1, an increase in voltage induces a relative shift between the feedback curve and the reflectance curve. This shift continues until the two curves become exactly tangent, forming a second intersection at Position 6. Due to the constraints imposed by the input voltage magnitude and negative feedback modulation, the operating point can only shift from Position 1 to Position 2. As the voltage increases further, the curves become tangent again. In this case, the operating point moves to the new tangent point at Position 3, while the intersection originally at Position 6 shifts to Position 4. Beyond this point, any additional increase in voltage causes the intersection at Position 3 to vanish. To stabilize the system output, the operating point transitions directly from Position 3 to Position 4, resulting in an abrupt jump in the output. Conversely, when the operating point is initially at Position 5 and the input voltage decreases, the operating point moves from Position 5 through Positions 4 and 6, then jumps directly to Position 2 before finally settling at Position 1. If the segment of the reflectance curve between Positions 1 and 3 is defined as the lower steady state and the segment between Positions 6 and 5 as the upper steady state, the relative shift between the feedback and reflectance curves can be interpreted as a switch between these two steady states, manifested as a bistable output signal. It should be noted that the applied voltage actually induces a shift in the Bragg wavelength of the fiber grating, meaning that the reflectance curve shifts (as shown in Supplementary Fig. 6). However, for the sake of clarity and consistency in description, this effect is represented here as a shift in the feedback curve. Both representations are functionally equivalent.

Fig. 2(c) illustrates the application scheme based on input optical power. Assuming the initial operating point of the system is at position 1, according to the theory of bistable systems, the slope of the feedback curve exhibits a downward trend as the optical power increases. Therefore, by increasing the optical power, the feedback curve can be gradually lowered until it first becomes tangent to the reflectance curve. At this moment, this point is marked as position 3, while the operating point moves from position 1 to position 2. If the optical power continues to increase, the feedback curve and the reflectance curve will undergo a second tangency, with the tangent point marked as position 4. The original tangent point 3 shifts to position 5, and the operating point moves from position 2 to position 4. A further increase in optical power will cause tangent point 4 to suddenly disappear. Based on the preceding analysis, to maintain stable output, the system must cause the operating point to transition directly from position 4 to position 5, resulting in an abrupt change in the system output. Similarly, when the operating point transitions from position 6 to position 1, this is achieved by reducing the optical power. The fundamental principle of this process is analogous to the description provided earlier and will not be reiterated here. Considering the difficulty of subsequent experimental design and practical operation, we primarily achieve bistable output by varying the input voltage.

Theoretically, as illustrated in Figs. 2(b)-(c), the distribution of the reflectance curve resembles a Gaussian function. By drawing an analogy from the expression for the Gaussian function, the expression for the reflectance curve function *R*[*V*(*t*)] can be derived as follows^6^:

$R\left[V(t)\right]=\alpha\cdot\exp\left\{\frac{-\left[V(t)-V_{max}\right]^{2}}{2w_{r}^{2}}\right\}+R_{min}$ (S19)

Here, *α* represents the optical path connection loss, *R_min_* is the minimum reflectivity, *V*(*t*) denotes the magnitude of the input PZT voltage, *V_max_* is the PZT voltage corresponding to the maximum reflectivity (at which the incident wavelength *λ* = *λ*_0_, with *λ*_0_ being the FBG reflection center wavelength), and *w_r_* is the standard deviation of the Gaussian approximation of the reflectivity curve. The total PZT voltage *V*(*t*) consists of the external voltage *V_ext_*(*t*) and the feedback voltage *V_f_*(*t*). The external voltage *V_ext_*(*t*) represents all voltages externally applied to the PZT. It can be either a DC bias voltage *V_d_* for hysteresis curve calibration (see Materials and methods section in main text), or the sum of the demodulated voltage *V_ac_*(*t*) and the injected noise *V_noise_*(*t*) for practical sensing (see Supplementary Note 1). Therefore, the voltage *V*(*t*) applied to the PZT can be expressed as:

$V(t)=V_{ext}(t)+V_{f}(t)$ (S20)

where *V_f_*(*t*) is the feedback voltage generated by the reflected optical power *I_B_*(*t*) through PD2 (see Fig. 3(a) in main text), given by:

$V_{f}(t)=\eta K_{1}K_{2}MI_{B}(t)$ (S21)

Here, *η* is the photoelectric conversion coefficient of PD2, *K*_1_ and *K*_2_ are the splitting ratios of the coupler, and *M* is the amplification factor of PD2. In this scheme, *K*_1_ = *K*_2_ = 0.5. Assuming a constant input optical power *I_in_* from the source, the reflected optical power is:

$I_{B}(t)=I_{in}\cdot R\left[V(t)\right]$ (S22)

Substituting Eq. (S22) into Eq. (S21) and then into Eq. (S20) yields the second functional relationship for the reflectance curve:

$R\left[V(t)\right]=\left[V(t)-V_{ext}(t)\right]\cdot\frac{1}{\eta K_{1}K_{2}MI_{in}}$ (S23)

Combining Eqs. (S19) and (S23), the intersection point of the two represents the actual operating point of the hybrid optoelectronic feedback system. The steady-state equation Ψ_0_[*V*(*t*)] of the FBG feedback loop is therefore:

$\Psi_{0}\left[V(t)\right]=V(t)-GI_{in}R\left[V(t)\right]=V_{ext}(t)$ (S24)

where *G* = *ηK*_1_*K*_2_*M* is the feedback gain coefficient. In the static limit *V_ext_*(*t*) = 0, Eq. (S24) has three distinct real roots:

$V_{1}<V_{2}<V_{3}$ (S25)

where *V*_1_ and *V*_3_ correspond to the two stable operating points and *V*_2_ to the unstable point. By varying the external input voltage or the input optical power, bistable output can be achieved. The three roots *V*_1_,*V*_2_,*V*_3_ define an effective bistable potential. Let:

$x(t)=\kappa\left[V(t)-\bar{V}\right],\bar{V}=\frac{V_{1}+V_{2}+V_{3}}{3}$ (S26)

where *κ* is a scaling factor converting the voltage to the system state variable. For notational simplicity, *x* is used as the independent variable in the Taylor expansion, while its time dependence enters through *V*(*t*). By expanding steady-state equation Ψ_0_[*V*(*t*)] around the three roots, we obtain:

$\Psi_{0}(x)\approx a_{eff}x-b_{eff}x^{3}+c_{eff}$ (S27)

where *a_eff_*, *b_eff_*, and *c_eff_* are the effective parameters determined by the three roots. These three parameters are not fixed constants. They depend on the local shape of the reflectance curve around the three roots and can be expressed as:

$\left\{\begin{matrix} a_{eff}=\frac{1}{\kappa}\cdot\left.\frac{d\Psi}{dV}\right|_{V=\bar{V}} \\ b_{eff}=-\frac{1}{6\kappa^{3}}\cdot\left.\frac{d^{3}\Psi}{dV^{3}}\right|_{V=\bar{V}} \\ c_{eff}=\Psi_{0}(\bar{V}) \end{matrix}\right.$ (S28)

Physically, *a_eff_* and *b_eff_* play the same roles as *a* and *b* in the standard bistable potential of Eq. (2) of the main text, determining the well depth, well position, and barrier height of the effective potential. *c_eff_* represents the asymmetry induced by the detuning between the laser wavelength and the FBG center wavelength. When *λ* = *λ*_0_, *c_eff_* = 0. The corresponding effective bistable potential is obtained by integrating −Ψ_0_(*x*) with respect to *x*:

$U_{eff}(x)=-\int\Psi_{0}(x)dx=-\frac{a_{eff}}{2}x^{2}+\frac{b_{eff}}{4}x^{4}-c_{eff}x$ (S29)

In the symmetric operating condition (the incident wavelength *λ* = *λ*_0_), *c_eff_* = 0, and Eq. (S29) reduces to the symmetric bistable potential *U*(*x*) in Eq. (2) of the main text. Taken together, these relations establish a continuous physical link from the external voltage to the effective bistable potential. The external voltage and the feedback voltage form the total PZT voltage, which shifts the FBG Bragg wavelength, modifies the reflectivity, and generates the feedback voltage that defines the effective bistable potential. Crucially, the laser wavelength *λ* and the input optical power *I_in_* do not enter the MGSR formulas directly. Instead, they modulate the effective potential parameters *a_eff_*, *b_eff_*, and *c_eff_* through the reflectance curve *R*[*V*(*t*)]. Varying *λ* shifts *V_max_* along the voltage axis, changing the positions of the three roots and thereby altering *a_eff_*, *b_eff_*, and *c_eff_*. Varying *I_in_* changes the feedback gain *GI_in_*, which modifies the slope of the feedback curve and thus reshapes the effective potential. This is the physical origin of the optical tunability of the MGSR sensing system. Details regarding the actual modulation effects of optical parameters on the MGSR sensing system can be found in Supplementary Note 5.

We now connect the FBG-generated potential to the MGSR theory developed in Supplementary Note 1. During practical sensing, the total external voltage is decomposed into the periodic driving signal *S*(*t*) ≡ *V_ac_*(*t*) from the hydrophone demodulation and the injected noise voltage *V_noise_*(*t*) from the DAQ card. The injected noise voltage serves as the stochastic driving term in the Langevin equation, denoted as *N*(*t*) ≡ *V_noise_*(*t*). Thus, *V_ext_*(*t*) = *S*(*t*)+*N*(*t*). Substituting this into Eq. (S24) and following the same procedure as in Supplementary Note 1, the effective bistable potential becomes time-dependent:

$U_{eff}(x,t)=-\frac{a_{eff}}{2}x^{2}+\frac{b_{eff}}{4}x^{4}-\left[c_{eff}-S(t)\right]\cdot x$ (S30)

where the linear term represents the combined effect of the intrinsic asymmetry *c_eff_* and the external driving *V_ext_*(*t*). The instantaneous barrier height of this tilted potential is:

$\Delta U_{eff}(t)=\Delta U_{eff,0}\cdot\left\{1-\left[\frac{c_{eff}-S(t)}{A_{c,eff}}\right]^{2}\right\}^{\frac{3}{2}}$ (S31)

where Δ*U_eff_*_,0_=*a_eff_*^2^/(4*b_eff_*) is the static barrier height of the FBG-generated potential, $A_{c,eff}=\sqrt{4a_{eff}^{3}/(27b_{eff})}$ is the effective critical amplitude. The corresponding instantaneous Kramers escape rate for the MGSR sensing system is therefore:

$r_{k,eff}(t)=\frac{1}{2\pi}\sqrt{U''_{eff}\left[x_{1}(t)\right]\left|U''_{eff}\left[x_{2}(t)\right]\right|}\exp\left[-\frac{\Delta U_{eff}(t)}{D}\right]$ (S32)

where $U''_{eff}(x)=-a_{eff}+3b_{eff}x^{2}$, and *x*_1_(*t*), *x*_2_(*t*) are the instantaneous roots of the tilted FBG potential. Following the same reasoning as in Supplementary Note 1, escape events occur predominantly at the instant when the barrier is lowest, when |*c_eff_* − *S*(*t*)| reaches its maximum. Evaluating Eq. (S32) at this instant and substituting the mechanical resonance response *A_eff_*(*f*) = *A*_0_·exp[−(*f* − *f_c_*)^2^/(2*w*^2^)], we obtain the frequency-dependent Kramers escape rate and optimal noise intensity for the MGSR sensing system:

$\left\{\begin{matrix} r_{k,eff}(f)=\frac{1}{2\pi}\sqrt{U''_{eff}\left[x_{1}(f)\right]\left|U''_{eff}\left[x_{2}(f)\right]\right|}\exp\left[-\frac{\Delta U_{eff}(f)}{D}\right] \\ D_{opt,eff}(f)=\frac{\Delta U_{eff}(f)}{\ln\left\{\frac{\sqrt{U''_{eff}\left[x_{1}(f)\right]\left|U''_{eff}\left[x_{2}(f)\right]\right|}}{4\pi f}\right\}} \end{matrix}\right.$ (S33)

where Δ*U_eff_*(*f*) = Δ*U_eff_*_,0_·{1− {[*c_eff_* − *A_eff_*(*f*)]/*A_c_*_,_*_eff_*}^2^}^3/2^ when |*c_eff_* − *S*(*t*)| reaches its maximum magnitude. This completes the theoretical framework of MGSR for the FBG-based sensing system. The acoustic pressure is first converted into a demodulated voltage through the acousto-structural coupling (Supplementary Note 11), this voltage serves as the periodic driving term in the Langevin equation, the FBG feedback loop generates an effective bistable potential whose parameters are optically tunable through *λ* and *I_in_*, and finally the MGSR theory predicts the frequency-selective enhancement and noise-robust behavior through Eqs. (S30)-(S33). This closes the theoretical gap between the generic SR equations and the FBG experimental implementation.

**Supplementary Note 4. Investigation of the impact of the MGSR sensing system delay on the Kramers escape rate matching condition**

In MGSR sensing systems, the closed-loop feedback loop inevitably introduces inherent delays originating from signal processing and actuator components. Although system delays are generally considered instantaneous on a macroscopic time scale, the phase shift induced by these delays may potentially compromise the matching conditions of stochastic resonance as the target signal frequency increases. To evaluate the actual influence of the inherent delay of the MGSR sensing system on the stochastic resonance performance, we conducted a systematic analysis and qualitative estimation.

The closed-loop feedback loop of the MGSR system mainly consists of the adder on the signal processing path, the demodulator, the photodetector, and the PZT. Among these components, the adder, implemented with a high-speed operational amplifier (OPA1678), has a response time on the microsecond scale, whereas the photodetector (OE-200-IN2) responds on the sub-microsecond scale. The demodulator employs a phase-generated carrier (PGC) modulation and demodulation algorithm on an embedded FPGA, whose processing delay is primarily governed by the order of the digital filters and the clock frequency, and remains on the microsecond scale for typical fiber-optic sensing systems. As for the PZT, it exhibits an inherent mechanical response time under external voltage excitation, and the response time of PZT-based components is typically on the microsecond scale^7^. Accordingly, the total closed-loop processing delay is estimated to be on the microsecond scale.

It should be noted that the data acquisition card is primarily used in this system to generate noise signals and inject them into the feedback loop. Since the phase of the noise signal is completely random, its statistical distribution characteristics are essentially unaffected by the delay of the data acquisition card. Therefore, this delay is not included in the delay estimation of the closed-loop feedback loop.

Meanwhile, classical stochastic resonance theory indicates that the synchronization between the system output and the input signal relies on the statistical matching condition that the Kramers escape rate *r_k_* and the input signal frequency *f* must satisfy *r_k_* = 2*f*. This matching condition is established based on the transition probability in statistical physics, rather than the strict locking of the instantaneous phase. Crucially, the maintenance of this statistical condition presupposes that the input signal's periodic trend remains intact. Within the primary frequency band investigated in this study (90 Hz to 180 Hz), the signal period is on the order of milliseconds, whereas the total closed-loop processing delay is on the order of microseconds. The delay therefore accounts for only a negligible fraction of the signal period, and the corresponding phase lag is far below the critical 180° threshold that would cause period confusion. Consequently, the macroscopic periodicity of the driving signal is fully preserved. Under this precondition, since the noise signal itself has a completely random phase distribution, the system is inherently insensitive to such slight shifts in the absolute phase of the input signal and solely needs to provide a statistical response to the intact macroscopic periodic driving force. Therefore, the influence of the inherent delay on the Kramers escape rate matching condition is essentially negligible.

**Supplementary Note 5. Effect of variations in optical wavelength and optical power on the output of MGSR sensing system**

Based on the analysis, variations in the frequency and amplitude of the input signal influence the efficacy of stochastic resonance processing. Additionally, analysis of Fig. 2(c) indicates that varying the optical power can also affect the outcome. Moreover, since altering the external input voltage effectively adjusts the relative detuning between the Bragg center wavelength of the fiber grating and the source wavelength, it follows that changing the source wavelength similarly impacts the stochastic resonance output. To investigate whether variations in optical wavelength and power significantly influence the stochastic resonance effect, we established default conditions at a wavelength of 1549.90 nm and an optical power of 1.8 mW. An input signal with a frequency of 80 Hz and an amplitude of 100 mV was used as the test subject. Under varying noise standard deviation conditions, the optical wavelength and power were sequentially varied. Finally, the output SNR after stochastic resonance processing was measured, and the results are presented in Supplementary Figs. 7(a)-(b).

Supplementary Fig. 7(a) illustrates the SNR variation under different optical wavelengths and noise intensities, with a fixed optical power of 1.8 mW. At a given noise intensity (*σ* = 2), as shown in the inset on the right side of Supplementary Fig. 7(a), increasing the wavelength from 1549.85 nm to 1549.95 nm alters the dwell time distribution of the operating point between the two stable states. Specifically, at 1549.85 nm, the operating point resides longer in the lower stable state, whereas at 1549.95 nm, it remains predominantly in the upper stable state. In contrast, at 1549.90 nm, the dwell time is approximately equally distributed. Consequently, compared to the balanced bistable output at 1549.90 nm, those obtained at 1549.85 nm and 1549.95 nm are less favorable for inducing stochastic resonance. For a given wavelength, although the highest SNR of 24.90 dB is achieved at 1549.85 nm, the SNR exhibits a monotonic decrease with increasing noise intensity, indicating that stochastic resonance does not occur under this condition. Instead, the elevated noise suppresses the system's ability to detect the input signal. At 1549.90 nm and 1549.95 nm, the SNR variations both exhibit stochastic resonance. However, as established above, the bistable output is significantly more stable at 1549.90 nm, rendering the stochastic resonance performance at this wavelength superior to that at 1549.95 nm. Thus, analysis of the relationship between optical wavelength and noise intensity reveals that the wavelength primarily governs the dwell time of the operating point in each stable state. This trend is consistent with the theoretical prediction in Supplementary Note 3, where the wavelength detuning controls the asymmetry parameter *c_eff_*, and *c_eff_* = 0 yields the optimal condition for stochastic resonance.

Supplementary Fig. 7(b) illustrates the variation in SNR obtained under different optical powers and noise intensities at an optical wavelength of λ = 1549.90 nm. For a constant noise intensity, varying the optical power induces two concurrent modifications in the bistable output: a change in output amplitude and a shift in the transition frequency of the operating point. The right inset in Supplementary Fig. 7(b) displays the time-domain output signals for a noise standard deviation of 2. As the optical power increases from 0.6 to 3 mW, the output amplitude progressively increases, and the waveform evolves from exhibiting subtle bistability to displaying a clear bistable waveform, followed by a reduction in waveform density. This behavior is attributed to the reduction in the slope of the feedback curve with increasing optical power, as analyzed in Fig. 2. This alters the number of intersection points between the feedback and reflectance curves, thereby modifying the transition behavior of the operating point. Under a periodically varying input voltage and constant noise intensity, an optical power of 0.6 mW results in a relatively steep feedback slope, resulting in a low probability of two intersection points between the feedback and reflectance curves. Consequently, the operating point remains confined to a single region, and bistable output is challenging to observe. At 1.8 mW, the periodic input voltage becomes sufficient to induce a transition in the operating point, enabling the output of a bistable waveform. Further increasing the power to 3 mW increases the feedback slope excessively, raising the input voltage required for the operating point transition and ultimately reducing the density of the bistable output waveform. Extending this analysis to consider the effect of varying noise intensity at a fixed optical power reveals the following: at 0.6 mW, the SNR decreases monotonically with increasing noise intensity, indicating the absence of stochastic resonance. At 3 mW, the SNR increases slowly with noise intensity, suggesting that the peak SNR has not yet been reached. Only at an optical power of 1.8 mW does the SNR reliably exhibit stochastic resonance under appropriate voltage regulation. Thus, analysis of the interplay between optical power and noise intensity demonstrates that variations in optical power influence the stochastic resonance processing effect through three primary mechanisms: output amplitude, operating point transition dynamics, and the system's capacity to generate stochastic resonance. These observations agree with the theoretical framework in Supplementary Note 3, in which the optical power modulates the feedback gain and thus the effective potential parameters *a_eff_* and *b_eff_*.

It should be noted that modulating the optical wavelength and power essentially adjusts the width of the hysteresis window. This is functionally equivalent to altering the position of the potential barrier in the bistable system, enabling the system to adapt to the prevailing noise environment. Simulations of the hysteresis window, obtained by disregarding the specific input and output of the system, are presented in Supplementary Figs. 7(c)-(d), illustrating that variations in optical wavelength shift the position of the hysteresis window, while changes in optical power alter its width. Consequently, optimal values for the optical power and wavelength exist, at which the stochastic resonance effect is maximized. Considering this mechanism, in conjunction with the MGSR discussed in the main text, the proposed stochastic resonance sensing system is expected to achieve a higher SNR gain.

**Supplementary Note 6. Workflow of the MGSR sensing system for practical detection tasks.**

In the main text, the structural composition and working principle of the MGSR sensing system have been clarified. To apply this system to practical detection tasks, we have devised a detection scheme based on the preceding analysis, as shown in Supplementary Fig. (8). The workflow of the MGSR sensing system is described as follows.

The workflow is divided into four stages, namely Prior Information Acquisition, MGSR Establishment, Dynamic Optimization, and Real-time Verification. In the Prior Information Acquisition stage, we pre-acquire the frequency response of the target sensor, the properties of the fiber Bragg grating (including reflectivity and central reflection wavelength), and the power spectral density of the ambient noise. This confirms the mechanical resonance frequency of the sensor and preliminarily estimates the initial values of the optical wavelength and power as well as the intensity of the ambient noise. Upon entering the MGSR Establishment stage, we set the initial optical wavelength *λ* and optical power *P* and obtain the optical hysteresis curve. By assessing whether a clear bistable transition phenomenon is present, we ensure that the system can output a stable bistable signal under the current parameters. If the bistable condition is not met, we revert and reset *λ* and *P*. Subsequently, we perform a preliminary analysis of the acquired time-domain and frequency-domain signals and calculate the signal-to-noise ratio of the target signal in the initial state, which serves as the baseline for subsequent optimization.

Based on the MGSR establishment, the system enters the Dynamic Optimization stage. We adopt a dual-layer nested search scheme in this stage, where the outer layer performs an optical power sweep and the inner layer performs an optical wavelength scan under a fixed optical power. After each scan, we record the highest output SNR achievable for the current parameter combination and compare it with the optimal SNR retained from the previous search round to calculate the SNR gain ΔSNR. When ΔSNR is greater than *ε* (where *ε* is the convergence threshold and *ε* is greater than 0), it indicates that the current parameter combination provides an effective improvement over the previous round. We update and retain the current optimal optical power and wavelength and proceed to the next round of sweeping to achieve continuous optimization. Conversely, when ΔSNR is less than or equal to *ε*, it indicates that the SNR gain has reached saturation. We consider the current parameters to be optimal, fix the optical power and wavelength, and record the peak SNR, denoted as SNR_max_, which serves as the basis for subsequent judgments on whether significant changes in ambient noise intensity have occurred.

In the Real-time Verification stage, once the optimal optical parameters are fixed, the system extracts the target signal under the current optical parameter settings and calculates the current real-time SNR, denoted as SNR_c_. If the absolute difference between the peak SNR and the current SNR satisfies the condition that the absolute value of SNR_max_ minus SNR_c_ is less than or equal to *ε*, it indicates that the ambient noise has not drifted significantly, and the system maintains the current parameter settings to achieve stable extraction. Conversely, if the absolute difference exceeds the threshold *ε*, it indicates that the ambient noise has drifted significantly, leading to system mismatch. In this case, we return to the Dynamic Optimization stage and restart the dynamic optimization process until new optimal optical parameters are fixed again, ultimately achieving real-time extraction of target signals by the MGSR sensing system under varying noise environments.

**Supplementary Note 7. Broader application potential of the stochastic resonance system**

Due to the structural independence between the sensing end and the bistable signal output end within the stochastic resonance system, beyond its applications in underwater acoustics, the system holds significant potential for cross-domain applications. Its scope of use can encompass all scenarios involving acoustic or vibration sensing, such as the four application contexts mentioned in the main text: pipeline leakage early warning, acoustic gravity wave observation, seismic wave monitoring, and aircraft identification. In the following sections, we will proceed with these four case studies to analyze sequentially how the stochastic resonance system can be applied to the aforementioned scenarios.

1. Early Warning of Pipeline Leakage

In water distribution systems (WDS), leakage constitutes the primary source of water loss in urban supply networks. However, conventional acoustic detection methods suffer from severe susceptibility to ambient noises, including fluid flow, vehicular traffic, and construction activities, which substantially compromises localization accuracy. The stochastic resonance system we report addresses this limitation by transforming detrimental environmental noise into a critical facilitator for signal amplification. By integrating existing fiber-optic sensors employed for pipeline leakage monitoring with our system, weak leak signals completely submerged in strong background noise can be effectively extracted and enhanced. This hardware-based approach directly mitigates the environmental noise interference challenge identified in previous WDS leakage detection studies^8^, offering a robust solution for precision leak localization in complex pipeline networks without recourse to computationally intensive post-processing algorithms.

2. Observation of Acoustic-Gravity Wave

Underwater moving targets, such as unmanned underwater vehicles (UUVs), inevitably generate acoustic-gravity waves characterized by low-frequency content and minimal attenuation, making them promising candidates for long-range detection^9^. However, their inherently low amplitude renders them highly susceptible to interference from natural environmental noise in practical detection scenarios. Our stochastic resonance system exhibits a pronounced amplification effect for low-frequency, low-amplitude signals, thereby substantially enhancing the detection and identification capabilities for underwater moving objects and making it particularly suitable for acoustic-gravity wave detection.

3. Monitoring of Seismic Waves

Seismic signals can propagate over long distances in the ocean. However, their spectral features are highly susceptible to contamination by ambient noise^10^. Conventional methods analyze the polarization characteristics of input signals in the time or frequency domain to separate seismic waves from noise, but they often suffer from insufficient frequency resolution under low signal-to-noise ratio conditions. The stochastic resonance system we proposed can serve as an effective front-end preprocessing module prior to conventional polarization analysis. By coupling seismo-acoustic signals with ambient noise and exploiting stochastic resonance, the polarization characteristics of target line spectra can be enhanced. This preprocessing strategy yields more stable and reliable extraction of seismic signals than conventional filtering methods alone, particularly when signal amplitudes approach the minimum detection threshold of the sensor.

4. Recognition of Aerial Vehicle

Helicopter acoustic signatures exhibit distinctive harmonic characteristics during far-field propagation, yet conventional detection methods experience substantial performance degradation in low signal-to-noise ratio environments^11^. The stochastic resonance system reported herein addresses this challenge by enabling the significant enhancement of weak harmonic signals received by microphone arrays under appropriate noise intensity conditions prior to conventional beamforming processing. This approach maintains algorithmic robustness while improving the resolution of direction-of-arrival estimation for helicopter acoustic signals, making it particularly suitable for low-energy harmonic components and non-stationary noise environments.

In summary, the stochastic resonance system developed in this work addresses critical bottlenecks in multiple sensing domains operating under strong background noise by virtue of its real-time processing capabilities and compatibility with all-optical sensing architectures. This integration paves the way for implementing simple, effective, and real-time stochastic resonance sensing across a wide range of application scenarios.

**Supplementary Note 8. Hybrid Packaging and Integrated Application of the MGSR Sensing System**

Currently, the MGSR sensing system is mainly implemented with discrete optical and electronic components, which inevitably increases deployment cost and spatial footprint, which hinders its large‑scale deployment in real‑world applications. Nevertheless, the underlying architecture of the MGSR sensing system essentially consists of fibre‑optic links and certain electronic components, which makes it naturally suitable for hybrid packaging and integration. In particular, with advances in chip-scale photonic integration, the on-chip integration of photonic components such as micro resonators, wavelength-division multiplexers, and photodetectors has been successfully demonstrated. Tan et al. integrated the dual-soliton microcomb with an on-chip arrayed waveguide grating, photodetectors and an FPGA control unit into a compact module, achieving on-chip multiplexing and demultiplexing of multi-wavelength channels^12^. Similarly, Li et al. used two on-chip micro ring resonators to generate Kerr soliton microcombs with different repetition frequencies and extracted specific comb lines via on-chip filters for parallel gas sensing^13^.

These examples fully demonstrate that key steps from light source generation to signal demodulation already have feasible chip‑scale integration solutions, and chip‑scale sensing systems are gradually approaching the maturity required for practical mission deployment. Given these technological advances, establishing a compact and packageable MGSR sensing system has become a feasible and reliable route towards practical applications. According to the underlying architecture of the current MGSR sensing system, optimization of system integration can be pursued from the following three aspects.

First, at the optical link level, the bistable feedback structure based on FBG can be miniaturized via a waveguide Bragg grating on a silicon photonic platform. Meanwhile, optical components such as light sources, circulators and couplers can also be integrated on chip using silicon‑based platforms. Such integration will substantially reduce system size and cost while preserving the core advantages of the MGSR sensing system, including its all‑optical front end and immunity to electromagnetic interference. Notably, the light source itself represents a promising direction for further enhancing the MGSR sensing system. Although the coherently parallel detection scheme based on dual Kerr soliton microcombs recently demonstrated by Li et al. differs from the current MGSR scheme that relies on single‑channel FBG feedback, their chip‑scale integrated light source and phase‑locking technology offer a valuable reference for improving light source quality and reducing phase noise in future MGSR systems^14^. As discussed in the main text, replacing the single FBG with a fibre Bragg grating array, in which multiple FBGs with distinct central wavelengths are serially written along the fibre to match the different comb lines of the microcombs, would allow each comb line to drive its own FBG feedback loop, thereby creating parallel stochastic resonance processing channels. This approach fully exploits the multi‑wavelength coherence of the optical comb while preserving the bistable operating principle of the FBG feedback mechanism, providing a new technological pathway for the MGSR sensing system to process broadband acoustic signals.

Second, at the electronic component level, elements such as PZT and adders can be integrated via MEMS platforms and hybrid‑packaged with the aforementioned silicon‑based structures. Third, at the sensor end, while retaining the existing hydrophones, micro‑ and nano‑sensing structures fabricated by MEMS‑based processes or emerging techniques such as 3D nanoprinting can be adopted as signal receivers for the MGSR system. By hybrid‑packaging the integrated light sources, passive optical devices, electronic components and miniaturized sensors, it is expected to ultimately achieve integrated integration of the MGSR sensing system at the levels of sensing, transmission and computation.

Therefore, the above integration strategy is expected to significantly reduce the deployment cost and spatial footprint of the MGSR sensing system, thereby promoting its large‑scale application in practical detection missions. In addition, the integrated MGSR system will also provide specific technical references for the development of other stochastic resonance systems toward fully integrated, field‑deployable sensors.

**Supplementary Note 9. Regulation strategy for FBG thermal sensitivity**

In the main text, we elaborated on the role of FBG in the MGSR sensing system, but also noted their inherent thermal sensitivity, where the central reflection wavelength drifts with temperature variations, thereby limiting the applicable range of the MGSR sensing system. Therefore, systematic temperature compensation strategies are essential to support the long-term field deployment of the MGSR sensing system in the future. Li et al. addressed the issue of FBG temperature compensation by proposing a scheme that maintains a constant temperature using a thermoelectric cooler (TEC)^13^. The TEC utilizes the Peltier effect, where a direct current drives a PN junction composed of two different semiconductor materials, enabling directional heat transfer at the junction interface to achieve heating or cooling, thereby maintaining a constant operating temperature and effectively suppressing slow thermal drift. Inspired by this, the MGSR sensing system can adopt a similar approach for FBG temperature control in future work, combined with the joint adjustment of optical power and wavelength (see Supplementary Note 5 for details), thereby ensuring stable detection performance during long-term, real-world deployments without frequent manual recalibration. Furthermore, the compact and portable nature of the TEC facilitates the future integration of the MGSR sensing system.

**Supplementary Note 10. Operating principle of the demodulator**

In the scheme for achieving stochastic resonance, the demodulator is a crucial component for analyzing the output of the hydrophone and generating an external output voltage signal. Its operating principle is based on the phase-generated carrier (PGC) modulation-demodulation algorithm. Specifically, the frequency of the demodulator's built-in light source is first subjected to cosine modulation, thereby inducing a substantial phase modulation within the fiber-optic interferometer. This causes the operating point to scan continuously between 0 and 2π. Subsequently, a stable phase value is output after processing by the demodulation algorithm. Based on the hydrophone's sensitivity, this demodulated phase value is then converted into a voltage signal *V_ac_*(*t*), which is superimposed onto the injected noise and the FBG feedback voltage to form the total PZT voltage *V*(*t*) in the optoelectronic feedback loop. The detailed operational process of the demodulator is as follows:

In the proposed scheme, the path difference between the sensing arm and the reference arm in the Michelson interferometer is denoted as *L_d_.* The light source integrated with the demodulator has a coherence length of *L*, and the refractive index of the fiber core is *n*_0_. Under the condition that *L* > 2*n*_0_*L_d_*, the interferometer can operate normally, and the resulting interferometric phase difference is:

 (S34)

Here, *f_opt_* is the optical frequency of the light source, and *c* is the speed of light in vacuum. This *ϕ_in_* should be distinguished from the acoustic-structural coupling phase *ϕ* in Eq. (S2) of Supplementary Note 1, which represents the initial phase of the demodulated acoustic signal. If the frequency of the light source is cosine-modulated at this point, the corresponding modulation phase change in the interferometer is:

 (S35)

Here, *f_mod_* represents the modulation frequency of the light source, and Δ*f* denotes the amplitude of the light source frequency modulation. If an acoustic signal is present in the environment at this time, after the optical signal returns to the interferometer, the raw voltage obtained via the photodetector can be expressed as:

 (S36)

Where *Z a*nd *B* are constants, *ϕ_s_* represents the phase change induced by the acoustic signal, and *ϕ*_0_ denotes the random phase fluctuation caused by environmental disturbances. Subsequently, by performing a Bessel function expansion on Eq. (S36) and extracting the phase of the measured acoustic signal in the form of quadrature signals, the following expression is obtained:

 (S37)

Among them, *J*_1_(*C*) is the first-order Bessel function, and *J*_2_(*C*) is the second-order Bessel function. Finally, through the arctangent algorithm, that is, by dividing the two expressions in Eq. (S37) and calculating their arctangent, the phase change of the measured acoustic signal can be obtained, with the formula as follows^15^:

 (S38)

Once the phase change of the acoustic signal under test is obtained, and if the current sensitivity of the hydrophone is known, the phase variation *ϕ_s_* can be converted into a voltage signal using the sensitivity relationship. This voltage signal is then superimposed onto the injected noise and the FBG feedback voltage, as described above. The expression for the fiber optic hydrophone sensitivity is as follows^16^:

 (S39)

Where *p* is the sound pressure, *S_h_*_0_ is the reference hydrophone sensitivity, and *V_ac_* is the output voltage signal of the demodulator. The derivation of how the acoustic pressure *p* produces the phase change *ϕ_s_* in Eq. (S36) is provided in Supplementary Note 11.

**Supplementary Note 11.** **Acousto-structural coupling of the push-pull fiber-optic hydrophone**

The phase change *ϕ_s_* in Eq. (S36) of Supplementary Note 10 originates from the external acoustic pressure through the acousto-structural response of the push-pull fiber-optic hydrophone. This note derives the transfer function *H_h_*(*f*) from the acoustic pressure to the demodulated voltage, following the model of the push-pull fiber-optic hydrophone^17^.

The push-pull fiber-optic hydrophone consists of two coaxial thin-walled elastic tubes (see Supplementary Fig. 4(a)). Tube 1 is in contact with water on its inner surface, and Tube 2 is in contact with water on its outer surface. Two fiber coils are wound on the outer surfaces of the two tubes, forming the two sensing arms of an unbalanced Michelson interferometer. Under a dynamic acoustic pressure Δ*P*, Tube 1 expands radially outward, while Tube 2 contracts radially inward. The radial boundary conditions on the two tubes are:

$\left\{\begin{matrix} T_{r,1}(r_{a,1})=-\Delta P,T_{r,1}(r_{b,1})=0 \\ T_{r,2}(r_{a,2})=0,T_{r,2}(r_{b,2})=-\Delta P \end{matrix}\right.$ (S40)

where *r_a,m_* and *r_b,m_* are the inner and outer radii of Tube *m* (*m* = 1,2), and *T_r,m_* is the radial stress. Using the two-dimensional plane-strain model for thin-walled cylinders, the radial and circumferential stresses on Tube *m* are:

$\left\{\begin{matrix} T_{r,m}=\frac{D_{m}}{r^{2}}+2E_{m} \\ T_{\theta,m}=-\frac{D_{m}}{r^{2}}+2E_{m} \end{matrix}\right.$ (S41)

where *E* and *ν* are the Young's modulus and Poisson's ratio of the tube material, and *D_m_*, *E_m_* are constants determined by the boundary conditions Eq. (S40) and Eq. (S41). By Hooke's law, the circumferential strain on the outer surface of Tube *m* is:

$\frac{\Delta r_{b,m}}{r_{b,m}}=\frac{1+\nu}{E}\left[-\frac{D_{m}}{r_{b,m}^{2}}+2E_{m}(1-2\nu)\right]$ (S42)

The phase change in the fiber wound on Tube *m* is:

 (S43)

where *n_eff_* is the fiber effective refractive index, *l_m_* is the fiber length wound on Tube *m*, *P*_11_ and *P*_12_ are the strain-optic coefficients, and *ν_f_* is the fiber Poisson's ratio. The total phase change of the push-pull structure is Δ*θ_total_* = 2(Δ*ϕ*_1_ − Δ*ϕ*_2_). The static analysis above neglects the dynamic response of the elastic tubes. In practice, the tubes are immersed in water, and the fluid loading introduces a frequency-dependent modulation of the circumferential strain. To capture this effect, the frequency-dependent response of the tubes must be considered. When fluid loading is considered, the circumferential strain becomes frequency-dependent:

$\frac{\Delta r_{b,m}}{r_{b,m}}(f)=\frac{1+\nu}{E}\left[-\frac{D_{m}}{r_{b,m}^{2}}+2E_{m}(1-2\nu)\right]\cdot F_{m}(f)$ (S44)

where *F_m_*(*f*) is the radial breathing mode response. Near the resonant frequency *f_c_*, *F_m_*(*f*) can be approximated by a second-order resonance:

$F_{m}(f)\approx\frac{1}{1-\left(\frac{f}{f_{c}}\right)^{2}+i2\xi\cdot\frac{f}{f_{c}}}$ (S45)

where *ζ* is the damping ratio. Taking the magnitude of Eq. (S45) and approximating the resulting resonance peak by a Gaussian form yields:

$\left|F_{m}(f)\right|\approx\exp\left[-\frac{(f-f_{c})^{2}}{2w^{2}}\right],w=\frac{f_{c}}{2.355Q}$ (S46)

where *Q* = 1/(2*ζ*) is the quality factor and *w* is the standard deviation of the Gaussian approximation. The transfer function from acoustic pressure to demodulated voltage is therefore:

$H_{h}(f)=A_{max}\cdot F_{m}(f)$ (S47)

where *A_max_* is the maximum response amplitude per unit acoustic pressure determined by the acoustic-structural coupling coefficient and the fiber length *l_m_*. The effective driving amplitude in Eq. (14) of the main text is:

 (S48)

where $\tilde{p}(r)$ is the acoustic pressure amplitude defined in Supplementary Note 1, and $A_{r}=A_{max}\cdot\left|\tilde{p}(r)\right|$ denotes the maximum response amplitude of the sensor at the resonant frequency under the actual acoustic pressure. This provides the physical origin of the Gaussian form used in the main text.

It should be noted that the Gaussian form of *F_m_*(*f*) is not a phenomenological fitting but a direct consequence of the sensor's physical structure and mechanical boundaries. Within the frequency range of interest in this work, the acoustic wavelength is two to three orders of magnitude larger than the sensor's transverse dimensions, so scattering is negligible and the incident pressure can be treated as uniformly loading the elastic tubes^18^. The push–pull structure also provides favorable acoustic impedance matching with water, as has been demonstrated for mandrel-type fiber-optic hydrophones employing low-acoustic-impedance resin materials^19^. The radial breathing mode frequency *f_c_* and quality factor *Q* are determined by the tube geometry, material properties, and boundary conditions at the end caps^20^. These physical factors collectively govern the resonance response, and the Gaussian form in Eq. (S46) is simply the near-resonance approximation to this physically determined frequency response.

**Supplementary Fig. 1. Simulation prediction of the output of a stochastic resonance system under non-mechanical resonance conditions.** (a) Signal-to-noise ratio curves obtained from the bistable stochastic resonance system model at an input signal amplitude of 0.465. (b) Prediction of the optimal noise intensity by the generalized Kramers escape rate theoretical model at an input signal amplitude of 0.465.

**Supplementary Fig. 2. Measurement of the fiber Bragg grating reflectivity curve.** (a) Experimental setup for measuring the fiber Bragg grating reflectivity. OPM: optical power meter; FBG: fiber Bragg grating; PZT: piezoelectric transducer; DCP: DC power supply. (b) Relationship between the fiber Bragg grating reflectivity and the input voltage. The input voltage refers to the applied DC voltage.

**Supplementary Fig. 3. Optical hysteresis curve test.** (a) Schematic of the fiber Bragg grating feedback structure. PD1 and PD2: photodetectors; DAQ: data acquisition card; OSC: oscilloscope. (b) Optical hysteresis curve. The curve comprises two segments: one segment corresponds to the DC voltage sweeping from -10 V to 10 V, and the other segment corresponds to the DC voltage sweeping from 10 V to -10 V.

**Supplementary Fig. 4. Frequency response curve measurement of the fiber-optic hydrophone.** (a) Structural composition of the fiber-optic hydrophone. (b) Experimental setup for frequency response curve measurement. FOH denotes the fiber-optic hydrophone, and AWG denotes the arbitrary waveform generator. (c) Output response curve of the fiber-optic hydrophone for input signal frequencies in the range of 20 Hz to 180 Hz.

**Supplementary Fig. 5. Validation of the Generalized Kramers Escape Rate Theory.** (a) Comparison of the optimal noise intensity predictions between the generalized and original theoretical models under non-mechanical resonance conditions. The input signal amplitude is 0.465. (b) Output signal-to-noise ratio of the bistable stochastic resonance system model when the input signal frequency ranges from 0.3 to 0.7 Hz. The reference lines correspond to the optimal noise intensity predictions of the two theoretical models under non-mechanical resonance conditions. (c) Comparison between the predicted and actual optimal noise intensities under non-mechanical resonance conditions. The average relative error of the predictions from the generalized formula is 3.1%, while that from the original formula is 22.9%. (d) Comparison of the optimal noise intensity predictions between the generalized and original theoretical models under mechanical resonance conditions, where the input signal amplitude is 0.697. The inset shows the current frequency response curve distribution. (e) Output signal-to-noise ratio of the bistable stochastic resonance system model when the input signal frequency ranges from 0.3 to 0.7 Hz. The reference lines are set under mechanical resonance conditions, analogous to (b). (f) Comparison between the predicted and actual optimal noise intensities under mechanical resonance conditions. The average relative error of the predictions from the generalized formula is 0.7%, while that from the original formula is 30.2%.

**Supplementary Fig. 6. Variation of the Bragg central wavelength of the fiber grating as a function of the applied voltage as it increases from 0 V to 10 V.**

**Supplementary Fig. 7. SNR curves under different light sources.** (a) SNR curves as a function of input optical wavelength and noise intensity. The right inset shows the variation of the time-domain signal obtained at different optical wavelengths. (b) SNR curves as a function of input optical power and noise intensity. The right inset shows the variation of the time-domain signal obtained at different optical powers. (c) Simulation of the effect of optical wavelength variation on the hysteresis window width. (d) Simulation of the effect of optical power variation on the hysteresis window width.

**Supplementary Fig. 8. Workflow of the MGSR sensing system.** The FBG properties include the reflectivity and the central reflection wavelength, whereas *ε* denotes the threshold for the SNR variation, which is utilized to determine whether the fluctuation of the current SNR remains within an acceptable range.

**Supplementary Table 1**

Definition of key variables and symbols and explanation of specialized terms used in the main text and supplementary materials.

| **Symbol** | **Description** |
| --- | --- |
| **Theoretical Analysis and Simulation of MGSR** | |
| *x* | System operating point position |
| *U*(*x*) | Bistable potential curve |
| *t* | Time |
| *S*(*t*) | Weak periodic excitation signal |
| *N*(*t*) | Noise signal |
| *δ*(⋅) | Dirac delta function |
| *a* | Bistable system constant |
| *b* | Bistable system constant |
| *A*_0_ | Amplitude of periodic excitation signal |
| *A_eff_*(*f*) | Effective driving amplitude after acousto-structural coupling |
| *A_r_* | Maximum response amplitude at mechanical resonance |
| *A_c_* | Critical input amplitude at which potential barrier vanishes |
| *f* | Input signal frequency |
| *f_c_* | Mechanical resonant frequency |
| *D* | Noise intensity |
| Δ*U* | Height of the bistable potential barrier |
| Δ*U*_0_ | Static barrier height |
| *r_k_* | Kramers escape rate |
| *r_kmax_* | Upper limit of Kramers escape rate |
| *w* | Standard deviation of the Gaussian mechanical resonance profile |
| *μ* | Effective barrier height modulation factor |
| $\tilde{x}$ | the dimensionless system state scaled by the characteristic length $\sqrt{a/b}$ |
| $\tilde{D}$ | the dimensionless noise intensity scaled by the static barrier height Δ*U*_0_ |
| $\tilde{A}_{0}$ | the dimensionless signal amplitude scaled by the critical amplitude *A_c_* |
| **Testing and Application of the MGSR Sensing System** | |
| *σ* | Standard deviation of noise |
| *λ* | Operating wavelength of tunable laser source |
| *P* | Optical power of laser source |
| *λ_B_* | Bragg central wavelength of FBG |
| *λ*_0_ | FBG reflection center wavelength |
| *R* | FBG reflectivity |
| *R_min_* | Minimum reflectivity of FBG |
| *R*(*V*) | Reflectance curve function |
| *w_r_* | Standard deviation of the Gaussian approximation of the reflectivity curve |
| *n_eff_* | Effective refractive index of fiber core |
| *Λ* | Grating period of FBG |
| *V_d_* | DC bias voltage applied to PZT |
| *V_max_* | PZT voltage corresponding to maximum reflectivity |
| *V*(*t*) | Total PZT voltage |
| *V_ext_*(*t*) | External voltage applied to PZT |
| *V_f_*(*t*) | Feedback voltage from photodetector |
| *V_ac_*(*t*) | Demodulated voltage from hydrophone |
| *V_noise_*(*t*) | Injected noise voltage |
| *V_PD_* | Raw voltage from photodetector |
| *η* | Photoelectric conversion coefficient of PD2 |
| *K*_1_*, K*_2_ | Splitting ratios of the optical coupler |
| *M* | Amplification factor of photodetector PD2 |
| *I_in_* | Input optical power from the source |
| *I_B_* | Reflected optical power from FBG |
| *α* | Optical path connection loss |
| *G* | Feedback gain coefficient |
| *Ψ*_0_(*V*) | Intrinsic steady-state function of FBG feedback loop |
| *V*_1_*, V*_2_*, V*_3_ | Three roots of steady-state equation |
| $\bar{V}$ | Average of three roots |
| *κ* | Scaling factor converting voltage to state variable |
| *a_eff_, b_eff_, c_eff_* | Effective potential parameter |
| *U_eff_*(*x*) | Effective bistable potential |
| *A_c,eff_* | Effective critical amplitude |
| Δ*U_eff_,*_0_ | Static barrier height of FBG-generated potential |
| *r_k,eff_* | Effective Kramers escape rate |
| *D_opt,eff_* | Effective optimal noise intensity |
| **Phase-Generated Carrier (PGC) Modulation and Demodulation Theory** | |
| *L* | Coherence length of the light source |
| *L_d_* | Path difference between sensing and reference arms |
| *n*_0_ | Refractive index of the fiber core |
| *c* | Speed of light in vacuum |
| *f_opt_* | Optical frequency of the light source |
| *ϕ_in_* | Interferometric phase difference in the Michelson interferometer |
| *f_mod_* | Modulation frequency of the light source |
| Δ*f* | Amplitude of the light source frequency modulation |
| *ϕ_mod_* | Phase change induced by light source frequency modulation |
| *ϕ_s_* | Phase change induced by acoustic signal |
| *ϕ*_0_ | Random phase fluctuation from environmental disturbances |
| *V*_1_(*t*), *V*_2_(*t*) | Quadrature signals |
| *J*_1_(*C*), *J*_2_(*C*) | First- and second-order Bessel functions |
| *Z, B* | Constants in the photodetector voltage expression |
| *S_h_* | Fiber optic hydrophone sensitivity |
| *S_h_*_0_ | Reference hydrophone sensitivity |
| **Acousto-structural Coupling Theory of the Push-Pull Fiber-optic Hydrophone** | |
| *ℜ*[·] | Real part of the argument |
| *p* | Acoustic pressure |
| *r* | Spatial position vector |
| *T_r,m_* | Radial stress on tube *m* |
| *r_a,m_*, *r_b,m_* | Inner and outer radii of tube *m* |
| *E* | Young's modulus of the tube material |
| *ν* | Poisson's ratio of the tube material |
| Δ*r_b,m_/r_b,m_* | Circumferential strain on the outer surface of tube mm |
| Δ*ϕ_m_* | Phase change in the fiber wound on tube mm |
| *l_m_* | Fiber length wound on tube mm |
| *P*_11_, *P*_12_ | Strain-optic coefficients |
| *ν_f_* | Fiber Poisson's ratio |
| Δ*θ_total_* | Total phase change of the push-pull structure |
| *H_h_*(*f*) | Hydrophone transfer function |
| $\tilde{p}(r)$ | Acoustic pressure amplitude |
| *F_m_*(*f*) | Radial breathing mode response |
| *A_max_* | Maximum response amplitude per unit acoustic pressure |
| *Q* | Quality factor |
| *ζ* | Damping ratio |
| **Sensor Performance Metrics** | |
| SNR | Signal-to-noise ratio |
| **Definitions of Key Terms** | |
| MGSR | Mechanically-gated stochastic resonance |
| FBG | Fiber Bragg grating |
| PZT | Piezoelectric ceramic |
| PD1, PD2 | Photodetectors |
| OSC | Oscilloscope |
| AWG | Arbitrary waveform generator |
| DAQ | Data acquisition card |
| FOH | Fiber-optic hydrophone |

**Supplementary Table 2**

Detailed parameter configurations for each simulation and experimental case

| **Parameter category** | **Symbol** | **Value/Range** | **Note** |
| --- | --- | --- | --- |
| **Figure 1** | | | |
| Input signal frequency | *f* | 60Hz | Figs. 1(a) - (c) |
|  |  | 40Hz, 60Hz, 80 Hz | Figs. 1(e) - (h) |
| Input signal amplitude | $\tilde{A}_{0}$ | 0.232 | Fig. 1(a) |
|  |  | 0.046, 0.093, 0.232 | Figs. 1(b) - (c) |
|  |  | 0.465, 0.282 | Fig. 1(e) |
|  |  | 0.930, 0.126 | Fig. 1(f) |
|  |  | 0.134, 0.325 | Fig. 1(h) |
| Noise intensity | $\tilde{D}$ | 0.8 | Fig. 1(a) |
|  |  | 0.08 - 1.6 | Figs. 1(b) - (c), (e) - (h) |
| Mechanical resonance | *—* | *Q* = 1.80, *A_r_* = 0.32 | Figs. 1(e) - (d) |
|  |  | *Q* = 3.61, *A_r_* = 0.64 | Figs. 1(d) - (f) |
|  |  | None | Fig. 1(g) |
|  |  | *Q* = 2.40, *A_r_* = 0.224 | Fig. 1(h) |
| **Figure 3** | | | |
| Operating wavelength | *λ* | 1549.95nm | Figs. 3(b) - (d) |
| Optical power | *P* | 3mW | Figs. 3(b) - (d) |
| Input signal frequency | *f* | 80Hz | Figs. 3(b) - (d) |
| Input signal amplitude | *A*_0_ | 100mV | Figs. 3(b) - (d) |
| Noise standard deviation | *σ* | 1, 2.65, 3 | Figs. 3(b)-(c) |
|  |  | 1.0-3.0 | Fig. 3(d) |
| **Figure 4** | | | |
| Input signal frequency range | *f* | 90Hz - 180Hz | Figs. 3(a) |
|  |  | 60Hz, 80Hz, 105Hz | Figs. 3(b) - (c) |
|  |  | 140Hz | Figs. 3(c) - (d) |
| Input signal amplitude | *A*_0_ | 30mV | Fig. 3(a) |
|  |  | 100mV | Figs. 3(b) - (c) |
|  |  | 10mV, 20mV, 30mV | Figs. 3(c) - (d) |
| Noise standard deviation | *σ* | 3.0 | Fig. 3(a) |
|  |  | 1.0 - 3.0 | Figs. 3(b) - (e) |
| Operating wavelength | *λ* | 1549.95nm | Figs. 3(b) - (c) |
|  |  | 1549.90nm | Figs. 3(d) - (e) |
| Optical power | *P* | 3mW | Figs. 3(b) - (c) |
|  |  | 1.8mW | Figs. 3(d) - (e) |
| **Figure 5** | | | |
| Operating wavelength | *λ* | 1549.90nm | Figs. 3(c) - (f) |
| Optical power | *P* | 1.08mW | Figs. 3(c) - (f) |
| Input signal frequency | *f* | 140Hz | Figs. 3(c) - (d) |
|  |  | 90Hz - 180Hz | Figs. 3(e) - (f) |
| Input signal amplitude | *A*_0_ | 1mV | Figs. 3(c) - (f) |
| Noise source | *—* | Ocean ambient noise (Noise 1) | Figs. 3(c) - (d) |
|  |  | Ocean ambient noise  (Noise 1, 2, 3) | Figs. 3(e) - (f) |
| **Supplementary Figure 1** | | | |
| Input signal amplitude | $\tilde{A}_{0}$ | 0.465 | Sup. Figs. 1(a) - (b) |
| Noise intensity range | $\tilde{D}$ | 0.08 - 1.6 | Sup. Fig. 1(a) |
| Signal frequency range | *f* | 10Hz - 150Hz | Sup. Fig. 1(b) |
| **Supplementary Figure 2** | | | |
| DC voltage sweep range | *V_d_* | 0V - 10V | Sup. Fig. 2(b) |
| **Supplementary Figure 3** | | | |
| DC voltage sweep range | *V_d_* | -10V to +10V | Sup. Fig. 3(b) |
|  |  | +10V to -10V |  |
| **Supplementary Figure 4** | | | |
| Input signal frequency range | *f* | 20Hz - 180Hz | Sup. Fig. 4(c) |
| **Supplementary Figure 5** | | | |
| Input signal amplitude | $\tilde{A}_{0}$ | 0.465 | Sup. Figs. 5(a) - (c) |
|  |  | 0.094, 0.423, 0. 697 | Sup. Figs. 5(d) - (f) |
| Signal frequency | *f* | 0.1Hz - 0.8Hz | Sup. Figs. 5(a), (d) |
|  |  | 0.3Hz - 0.7Hz | Sup. Figs. 5(b) - (c),  (e) - (f) |
| Noise intensity | $\tilde{D}$ | 0 - 1.6 | Sup. Figs. 5(b), (e) |
| Mechanical resonance | *—* | None | Sup. Figs. 5(a) - (c) |
|  |  | *Q* = 3.0, *A_r_* = 0.48 | Sup. Figs. 5(d) - (f) |
| **Supplementary Figure 6** | | | |
| Applied voltage | *V_d_* | 0V, 5V, 10V | Sup. Fig. 6 |
| **Supplementary Figure 7** | | | |
| Operating wavelength | *λ* | 1549.85nm, 1549.90nm, 1549.95nm | Sup. Fig. 7(a) |
|  |  | 1549.90 nm | Sup. Fig. 7(b) |
| Optical power | *P* | 1.8 mW | Sup. Fig. 7(a) |
|  |  | 0.6mW, 1.8mW, 3 mW | Sup. Fig. 7(b) |
| Input signal frequency | *f* | 80Hz | Sup. Figs. 7(a) - (b) |
| Input signal amplitude | *A*_0_ | 100mV | Sup. Figs. 7(a) - (b) |
| Noise standard deviation | *σ* | 1, 2, 3 | Sup. Figs. 7(a) - (b) |

**Supplementary References**

1.	Jacob, S., Trigell, E., Mihaescu, M.*, et al.* Acoustic scattering in a small centrifugal compressor based on the use of linearized equations in a rotating frame. *Journal of Sound and Vibration* **544**, <https://doi.org/10.1016/j.jsv.2022.117315> (2023).

2.	McNamara, B. & Wiesenfeld, K. Theory of stochastic resonance. *Physical Review A* **39**, 4854–4869 <https://doi.org/10.1103/PhysRevA.39.4854> (1989).

3.	Lai, Z.-h. & Leng, Y.-g. Weak-signal detection based on the stochastic resonance of bistable Duffing oscillator and its application in incipient fault diagnosis. *Mechanical Systems and Signal Processing* **81**, 60–74 <https://doi.org/10.1016/j.ymssp.2016.04.002> (2016).

4.	Gammaitoni, L., Hänggi, P., Jung, P.*, et al.* Stochastic resonance. *Reviews of modern physics* **70**, 223–287 <https://doi.org/10.1103/RevModPhys.70.223> (1998).

5.	Monnai, T., Sugita, A. & Nakamura, K. Kramers theory in the relaxation dynamics of a tilted asymmetric periodic potential. *Physical Review E* **76**, <https://doi.org/10.1103/PhysRevE.76.031140> (2007).

6.	Guohui, L., Hongan, Y., Junqing, L.*, et al.* Optical bistability in tunable fiber laser using fiber Mach-Zehnder interferometer. *Chinese optics letters* **3**, <https://doi.org/10.1364/col.3.1.18> (2005).

7.	Premkumar, S., Shinde, S. D., Kumar, H. H.*, et al.*, Characterization of PZT multi-layer actuator, 1st International Symposium on Physics and Technology of Sensors (ISPTS-1), Pune, India, <https://doi.org/10.1109/ISPTS.2012.6260881> (2012).

8.	Guo, G., Yu, X., Liu, S.*, et al.* Novel Leakage Detection and Localization Method Based on Line Spectrum Pair and Cubic Interpolation Search. *Water Resources Management* **34**, 3895–3911 <https://doi.org/10.1007/s11269-020-02651-z> (2020).

9.	Dong, Q., Zhao, X., Huang, B.*, et al.* Low-frequency acoustic-gravity wave emission generated by the vortex caused by a moving submerged cylinder. *Applied Ocean Research* **147**, <https://doi.org/10.1016/j.apor.2024.103990> (2024).

10.	Han, X. & Piao, S.-c., A line spectrum extraction method of seismic-acoustic signal, Symposium on Piezoelectricity, Acoustic Waves, and Device Applications (SPAWDA), Jinan, China, <https://doi.org/10.1109/SPAWDA.2015.7364541> (2015).

11.	Yi, H., Chen, Y. & Huang, J., Aerial Acoustic Targeting Orientation Estimation Based on Deconvolved Conventional Beamforming, IEEE International Conference on Signal Processing, Communications and Computing (ICSPCC), Zhengzhou, China, <https://doi.org/10.1109/icspcc59353.2023.10400361> (2023).

12.	Tan, T., He, X.-Y., Chang, B.*, et al.* Biomimetic acoustic perception via chip-scale dual-soliton microcombs. *eLight* **5**, <https://doi.org/10.1186/s43593-025-00099-5> (2025).

13.	Li, Y.-W., Wang, Y.-C., Liu, Z.-H.*, et al.* Gas mapping based-on dual microcomb driven nanomaterial functionalized fiber Bragg grating string. *PhotoniX* **7**, <https://doi.org/10.1186/s43074-025-00225-z> (2026).

14.	Li, J.-T., Chang, B., Du, J.-T.*, et al.* Coherently parallel fiber-optic distributed acoustic sensing using dual Kerr soliton microcombs. *Science advances* **10**, <https://doi.org/10.1126/sciadv.adf8666> (2024).

15.	Hou, C., Zhang, J., Yuan, Y.*, et al.* Reliability Demodulation Algorithm Design for Phase Generated Carrier Signal. *IEEE Transactions on Reliability* **71**, 127–138 <https://doi.org/10.1109/tr.2021.3125068> (2022).

16.	Luo, Z., Yang, Y., Wang, Z.*, et al.* Low-frequency fiber optic hydrophone based on weak value amplification. *Optics Express* **28**, <https://doi.org/10.1364/oe.400373> (2020).

17.	Yang, Y., Luo, Z., Wang, Z.*, et al.* High-Performance Fiber Optic Interferometric Hydrophone Based on Push–Pull Structure. *IEEE Transactions on Instrumentation and Measurement* **70**, 1–13 <https://doi.org/10.1109/tim.2021.3096604> (2021).

18.	Baynes, A. B. & Godin, O. A. Scattering of low frequency sound by fluid and solid cylinders. *Journal of Sound and Vibration* **434**, 336–357 <https://doi.org/10.1016/j.jsv.2018.07.004> (2018).

19.	Fu, Y., Kabir, I. I., Yeoh, G. H.*, et al.* A review on polymer-based materials for underwater sound absorption. *Polymer Testing* **96**, <https://doi.org/10.1016/j.polymertesting.2021.107115> (2021).

20.	Yang, Y., Chang, T., Yu, M.*, et al.* Physics-Based Performance Optimization of Fiber-Optic Interferometric Hydrophone With Push–Pull Mandrel Structure. *IEEE Sensors Journal* **22**, 19346–19356 <https://doi.org/10.1109/jsen.2022.3201908> (2022).
